# pH-susceptibility of HLA-DO tunes DO/DM ratios to regulate HLA-DM catalytic activity

**DOI:** 10.1038/srep17333

**Published:** 2015-11-27

**Authors:** Wei Jiang, Michael J. Strohman, Sriram Somasundaram, Sashi Ayyangar, Tieying Hou, Nan Wang, Elizabeth D. Mellins

**Affiliations:** 1 Department of Pediatrics, Stanford University, Stanford, CA 94305, USA; 2 Stanford Program in Immunology, Stanford University, Stanford, CA 94305, USA

## Abstract

The peptide-exchange catalyst, HLA-DM, and its inhibitor, HLA-DO control endosomal generation of peptide/class II major histocompatibility protein (MHC-II) complexes; these complexes traffic to the cell surface for inspection by CD4+ T cells. Some evidence suggests that pH influences DO regulation of DM function, but pH also affects the stability of polymorphic MHC-II proteins, spontaneous peptide loading, DM/MHC-II interactions and DM catalytic activity, imposing challenges on approaches to determine pH effects on DM-DO function and their mechanistic basis. Using optimized biochemical methods, we dissected pH-dependence of spontaneous and DM-DO-mediated class II peptide exchange and identified an MHC-II allele-independent relationship between pH, DO/DM ratio and efficient peptide exchange. We demonstrate that active, free DM is generated from DM-DO complexes at late endosomal/lysosomal pH due to irreversible, acid-promoted DO destruction rather than DO/DM molecular dissociation. Any soluble DM that remains in complex with DO stays inert. pH-exposure of DM-DO in cell lysates corroborates such a pH-regulated mechanism, suggesting acid-activated generation of functional DM in DO-expressing cells.

The polymorphism of major histocompatibility complex (MHC) alleles enables their gene products to associate with a highly diverse set of peptides derived from both self and foreign antigens. For MHC class II molecules (MHC-II), which are expressed on antigen presenting cells (APC) and present peptides to CD4+ T cells, sequential intracellular interaction with accessory molecules, invariant chain (Ii) and HLA-DM (DM), regulates peptide loading. Nascent MHC-II α/β heterodimers in the endoplasmic reticulum (ER) assemble into complexes with Ii, which inhibits their interaction with other ligands. Ii also directs the intracellular trafficking of MHC-II-Ii complexes into the endosomal pathway, where proteases gradually trim Ii, leaving class II-associated Ii peptides (CLIP) bound to the MHC-II peptide-binding groove^1^. The polymorphic nature of MHC-II confers strong allelic dependence on the strength of CLIP binding^2^, especially at different endosomal pHs^3^. For most alleles, efficient exchange of CLIP for other peptides is dependent on the nonclassical, class II-like molecule, DM. DM has enzyme-like activity and enhances peptide exchange most efficiently at late-endosomal/lysosomal pHs (4.5–6.0)^4^. Through a pH-dependent, transient DM/MHC-II association^5^,^6^, DM stabilizes a peptide-receptive form of MHC-II^4^,^5^ and accelerates the dissociation of low affinity peptides (e.g., CLIP)^7^, thereby selecting high affinity peptides for presentation at the APC surface. This peptide editing function of DM contributes significantly to the immunodominance of certain epitopes over others^8^.

A second non-classical MHC-II molecule, HLA-DO (DO), is an inhibitor of DM function and is expressed in a subset of DM-expressing APC types, specifically B cells, some types of dendritic cells (DC) and thymic medullary epithelia^9^,^10^,^11^. The crystal structures of DM-DO and DM-MHC-II complexes show that DO and MHC-II bind DM similarly (Fig. 1a), and, together with kinetic data, argue strongly that DO acts as a competitive inhibitor^12^,^13^,^14^,^15^. The egress of DO from the ER and its intracellular transport to endosomal compartments requires tight association with DM^16^,^17^. Free DO has not been observed in cells. This suggests that DO/DM ratios are always ≤1 in endosomal compartments despite differential expression of DO among APC types^9^,^11^ and among maturation/activation states of DO-expressing cells^10^,^18^,^19^,^20^. Both DM and DO are residents of the very acidic lysosomes^16^,^21^,^22^, which significantly complicates the investigation of the pH-regulation of DM-DO function in cells.

It is known that DM activity can arise in DO expressing APC and is enhanced after activation of B cells or maturation of DO expressing DC. Several scenarios have been postulated, including that DM-DO complexes dissociate into separate DM and DO (2 heterodimers) or that DM-DO persists as a tetrameric complex that undergoes conformational change allowing DM activity. Direct evidence for either of these mechanisms is lacking, however.

Mechanistic studies have been pursued *in vitro*. The analysis of DM-DO regulated peptide exchange *in vitro* is also challenging, however, due to MHC allelic variation, DM-DO’s bi-molecular substrate (MHC-II/peptide), the instability of DO in the absence of DM^16^,^17^, and the pH sensitivities of peptide/MHC interactions^23^,^24^, DM/DR interaction^5^,^6^ and DM-catalyzed peptide exchange^4^ (Fig. 1a). One bottleneck is the lack of a robust biochemical method for pH analyses. Here, we optimized an immunoassay to differentiate the pH effects on class II peptide exchange, DM catalysis, and DO inhibition of DM. As the inhibitory function of DO relies on its association with DM, we also quantitatively determined the interaction between ectodomains of DM and DO at various pHs, using multiple techniques. Our approaches allowed us to elucidate the mechanism of pH-regulated DM-DO function.

## Results

### DM concentration is critical to the pH range of its catalytic activity

**Figure 1:**
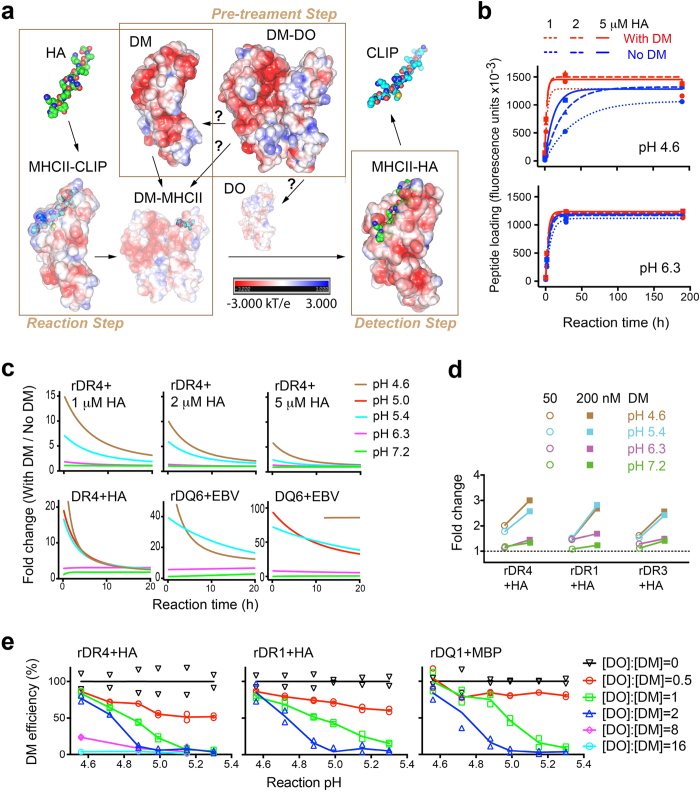
pH affects peptide exchange, DM catalysis and DO inhibition. (**a**) Immunoassays allow assessment of peptide loading in three separated steps each containing multiple pH-sensitive processes, denoted by arrows. Question marks indicate undefined mechanisms. Shown are electrostatic potential surfaces of each structure (PDB: 1HDM, 4I0P, 4FQX, 3PDO, and 1DLH). (**b**) Recombinant DR4 (5 nM) was incubated with indicated concentrations of HA_306–318_ peptide in the presence or absence of soluble DM (200 nM) at the indicated pH for different reaction times, followed by measurement of DR4-HA complexes quantified as fluorescence units and indicated as peptide loading. Data were fit to a kinetic model (see **Methods**) (**c**) Fold difference between peptide loading (HA_306–318_ to DR4 or EBV_490–503_ to DQ6, r: recombinant; f: full length) with and without DM at each pH was calculated (first 20 h), based on the fitted curves from (**b**) or (Supplementary Fig. 1). (**d**) HA loading to different DR allelic proteins with various concentrations of DM was carried out for 1.5 h at the indicated pH (detailed in Supplementary Fig. 2a) and was normalized to individual allelic baseline (corresponding pH conditions without DM) and expressed as fold change. (**e**) Peptide loading (HA_306–318_ to DR proteins or MBP_86–99_ to DQ1) at different reaction pHs was performed with various [DO]:[DM] (detailed in Supplementary Fig. 2c), normalized to corresponding reactions with DM alone after subtraction of individual allelic baselines, and represented as DM efficiency. [DO]:[DM] = 0 indicates DM only. The mean of duplicates is shown (**a**–**d**) or both replicates are shown (**e**). All data are representative of two experiments.

An ELISA-based immunoassay (See **Methods**) advances the study of pH effects on peptide loading, due to its ability to separate steps, such as acid pre-exposure, peptide loading reactions, and detection of nascent MHC-II/peptide products (Fig. 1a). Importantly, the neutralization of each peptide loading reaction before time-resolved fluorescence (TRF) based quantitation of MHC-II/peptide products ensures minimum pH influence on fluorescence measurements.

Unlike measures of reaction kinetics, the immunoassay captures peptide-exchange outcomes that are predominantly dependent on reaction time. We, therefore, first determined the optimal time frame for reliable measurements of pH-regulated catalysis of peptide loading. Based on a kinetic model (See **Methods**), we measured spontaneous and DM-catalyzed peptide exchange as a function of time at different pHs (Fig. 1b and Supplementary Fig. 1) and converted the DM effect to “fold change” (Fig. 1c). As pH differentially affects spontaneous peptide binding to different MHC-II alleles^23^,^24^, we used individual baselines for uncatalyzed peptide exchange to allow assessment of DM effect/fold change across alleles. Within 3 h of reaction, the fold change consistently decreased as pH increased, regardless of the substrate for peptide loading, e.g. affinity-purified recombinant proteins (rDR4, rDQ6) or corresponding full length molecules (fDR4, fDQ6) in cell lysates (Fig. 1c). This short reaction time also limited acid-induced loss of class II molecules (Supplementary Fig. 1). Notably, higher peptide concentrations attenuated the extent of DM effect without affecting its pH-dependence (Fig. 1c).

We next used the optimum time frame (≤2 h peptide loading reaction time was used henceforward) to evaluate the correlation between DM abundance and DM activity at different pHs. Consistent with previous results^4^, DM function was optimal at late-endosomal/lysosomal pH (5.4 or 4.6, respectively) and generally diminished above pH 6.3. However, higher concentration significantly increased DM activity at all tested pHs, including the less favorable pHs, 6.3 or 7.2 (Fig. 1d and Supplementary Fig. 2a), arguing that increasing DM concentration broadens the pH range of its catalytic function.

### DO/DM ratios control the pH range of efficient peptide exchange

The effective concentration of DM is influenced by the presence of its inhibitor, DO. Further, it is thought that pH influences DO regulation of DM. However, precise and quantitative analyses have been difficult, and data on pH effects have been conflicting. Affinity-purified DM-DO from cells never yields [DO]:[DM] > 1, and sometimes contains over 15% free DM^25^; this likely has contributed to previous inconsistencies regarding pH dependence of DO function^14^,^15^,^26^,^27^. To analyze DO effects on DM function across a pH range, we modified the ectodomain of DO, such that it can be expressed and purified alone. This stabilized DO variant (DO_v_) bears a single mutation in the α chain (αP11A), distant from the contact interface of DO and DM^13^, and functions equivalently to wild type DO^26^. We observed no effect on peptide exchange by DO_v_ alone at all tested pHs (Supplementary Fig. 2b). Consistent with our previous finding that DO was capable of inhibiting DM at pH 4.5–7.0 (ref. 26), DM catalysis of peptide exchange was inefficient at all tested pHs when [DO]:[DM] ≥ 8 (Fig. 1e). However, at lower [DO]:[DM], we observed efficient DM function (escape from DO inhibition) at acidic pH. Importantly, the pH range for efficient DM catalysis broadened as [DO]:[DM] decreased: (4.5–4.7) at ratio 2, (4.5–4.9) at ratio 1, and (4.5–5.5) at ratio 0.5 (Fig. 1e), consistent with the relationship we observed between DM abundance and pH range of DM activity. The correlation between [DO]:[DM] and pH range of efficient DM catalysis is MHC-II allele independent (Fig. 1e and Supplementary Fig. 2c). Given the critical importance of free DM levels to DM function, we next studied how DM escape from DO inhibition is regulated by pH and sought to determine whether this occurs by altering the interaction between the two proteins or by direct pH effects on DM or DO alone.

### The interaction between intact DO and DM is pH-independent

**Figure 2:**
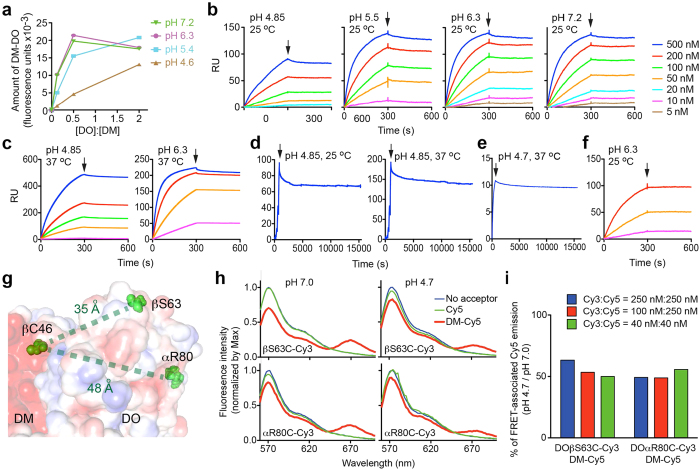
pH influences DO/DM interaction. (**a**) Different ratios of soluble DO_v_/DM proteins were incubated at the indicated pH for 1.5 h before the measurement of DM-DO_v_ complexes by capture ELISA (see **Methods**). (**b**,**c**) SPR (BIAcore) analysis of binding of DO_v_ injected at indicated concentrations to immobilized DM under indicated conditions. Arrows indicate the end of DO_v_ injection and the beginning of dissociation. (**d**) A single cycle mode with extended dissociation (>4 h after the arrow) was selected to monitor dissociation of DM-DO_v_ complexes formed on the BIAcore sensor at indicated conditions. (**e**) Dissociation of DM-DO_wt_ complexes analyzed by BLI (Octet OK) at the indicated condition. (**f**) Intact DO_v_ SEC-purified after pH 4.6 pulses (Supplementary Fig. 4) was analyzed by SPR for DM binding at the indicated condition and colored as in (**b**). (**g**) Illustration of sites (red spheres) used for mutagenesis and/or dye labeling, with interatomic distances as indicated. PDB code: 4I0P. (**h**) FRET analysis of donors: 250 nM of Cy3-labeled proteins (DO mutants-Cy3) or buffer, and acceptors: 250 nM of Cy5-labeled protein (DM-Cy5) or equivalent free Cy5 or buffer, at indicated pHs. Emission spectrum was normalized by the maximum (~570 nm) donor emission after background (acceptor alone) correction. (**i**) Integrals (the area under the curve) of FRET-associated Cy5 emission in (**h**) were calculated. Shown is the ratio of the integral at pH 4.7 to that at pH 7.0. The mean of duplicates is shown (**a**,**h**). Data are representative of two (**a**–**f**) or three (**h**) experiments. Temperature effects on BIAcore sensor surfaces besides pH effects significantly contributed to RU, but had no influence on measurements of *K*_*D, app*_ (Supplementary Table. 1).

We first used ELISA to measure the levels of DM-DO_v_ complexes formed *in vitro* at varied pH. When [DO]:[DM] ≤ 0.5, there was significantly less DM-DO_v_ detected at pH 4.6 than at other pHs tested; however, when [DO]:[DM] = 2 (i.e., DO_v_ was not limiting) DM-DO_v_ accumulation at pH 4.6 was close to the level observed at higher pHs (Fig. 2a), suggesting a pH-susceptible feature of DO rather than pH effects on DO/DM interaction.

We next measured real-time association/dissociation of DM-DO_v_ complexes. We used two label-free techniques, surface plasmon resonance (SPR) and bio-layer interferometry (BLI), to avoid any effects of pH on labeling agents. Notably, there was negligible complex dissociation observed across all tested pHs, at both RT and 37 °C, by SPR (Fig. 2b–d). Similar results were observed using BLI (Supplementary Fig. 3). The lack of dissociation was not merely due to the use of the stabilized DO_v_ mutant, because dissociation was not detected when wild type DM-DO_wt_ ectodomains were used in the BLI analysis (Fig. 2e). These results also support the notion that, once formed, the molecular interactions between DO and DM are pH resistant. In contrast, we observed reduced complex accumulation at acidic pH, consistent with DO itself being pH vulnerable. To test this idea further, we isolated the intact portion of acid-pulsed DO_v_ by size exclusion chromatography (SEC) (Supplementary Fig. 4); this fraction of DO_v_ maintained DM binding at pH 6.3 (Fig. 2f). To directly compare binding kinetics at different pHs, we estimated the apparent equilibrium dissociation constant *K*_*D, app*_ by assuming that the DO/DM interaction fits a slow dissociation model (see **Methods**). Although *K*_*D, app*_ increased significantly (>10 fold) when pH dropped from 7.2 to 4.7, the intact portion of acid-pulsed DO_v_ bound DM to nearly the same extent as non-treated DO_v_ (Supplementary Table 1), implying that pH does not affect the interaction of DM and DO_v_ as long as they are not denatured/destroyed by acid.

As another approach, we studied pH effects on DO/DM interaction using fluorescence resonance energy transfer (FRET) analysis. The observed distance (<100 Å) between DOβS63 or DOαR80 and DMβC46 in the DM-DO structure^13^ (Fig. 2g) predicts that binding of Cy3-labeled DO mutants (DO_v_βS63C and DO_v_αR80C) and Cy5-labeled DM will generate FRET^26^. FRET-associated Cy5 emission peak (670 nm) should decrease if inter-dye distance increases or less DM-DO forms. Mutation and labeling caused negligible interference on DM-DO functions (Supplementary Fig. 5). FRET-associated Cy5 emission between Cy3-labelled DO mutants and Cy5-labelled DM significantly decreased when the pH changed from 7.0 to 4.7 (Fig. 2h). The percent of pH-induced FRET reduction was consistently ~50% (Fig. 2i), likely reflecting an acid-dependent reduction in intact DO molecules rather than diminished binding of intact partners or chemical loss of dyes (Supplementary Fig. 6). Furthermore, when we kept [DO_v_βS63C] constant, FRET-associated Cy5 emission was almost identical as long as sufficient DM ([DM] > [DO_v_βS63C]) accepted all transferrable fluorescence energy from associated DO (Supplementary Fig. 6b). This concentration-independence is consistent with the interpretation that pH barely affects intact DO/DM interaction, with the exception of denaturing intact DO molecules.

### Acidic pH primarily denatures DO to tune DO/DM ratios

**Figure 3:**
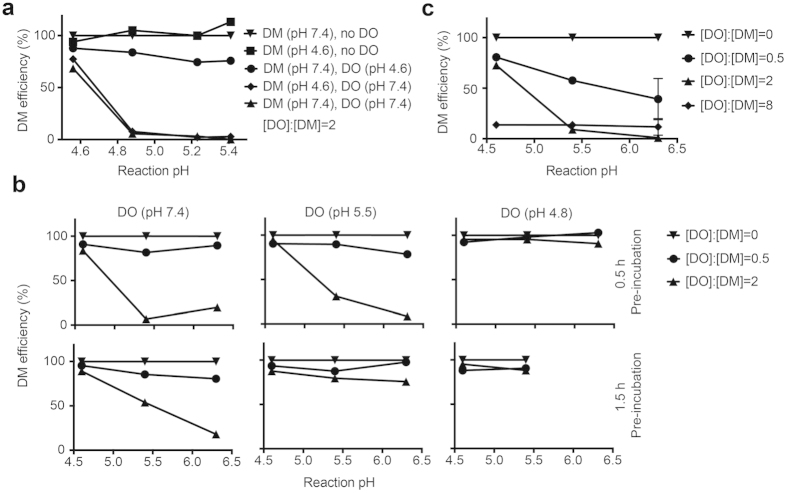
Immunoassays with acid pre-treated DM and DO. (**a**) DO_v_ (400 nM) or DM (200 nM) was pre-incubated at the pH as indicated in parenthesis, 4 °C for 4 days and then mixed with DR4 (5 nM) and HA_306–318_ (1.5 μM). HA loading at different reaction pHs was carried out at 37 °C for 1.5 h before the measurement of DR4-HA complexes. DM efficiency (%) was calculated as in Fig. 1e. (**b**) DO_v_ was pre-incubated at the pH as indicated in parenthesis, 37 °C for 0.5 or 1.5 h and then used at the indicated DO/DM ratios in the HA loading assay, described in (**a**). (**c**) Intact DO_v_ SEC-purified after pH 4.6 pulses (Supplementary Fig. 4) was used in the HA loading assay at the indicated ratios with DM. Error bars represent standard error of mean (SEM) for triplicates. Data are representative of two (**a**,**b**) or three (**c**) experiments.

To investigate whether, in addition to its effects on DO, pH also affects DM to promote escape of DM activity from DO inhibition, we pre-incubated DM or DO_v_ separately at pH 4.6, 4 °C for 4 days and compared them to untreated counterparts in peptide loading immunoassays. The moderate acid pre-treatment had limited effect on DM activity. However, it was sufficient to alter DO_v_, such that, unlike untreated DO_v_ (Fig. 1e), acid-treated DO_v_ allowed efficient DM action at pH > 4.9 when [DO]:[DM] = 2 (Fig. 3a). This alteration occurred faster at lower pHs and higher temperatures (Fig. 3a,b). Notably, after 0.5 h pre-incubation at pH 4.8, 37 °C, 2 fold excess of DO_v_ was unable to inhibit DM, even after being returned to pH 5.4 or 6.3 where untreated DO_v_ functions (Fig. 3b vs Fig. 1e). Different from the reversible pH-sensitivity described previously^14^, this acid-induced alteration of DO appears irreversible. In addition, we observed no difference in DM inhibition comparing the SEC-purified intact DO from pH 4.6 pre-treated DO to untreated DO (Fig. 3c vs Fig. 1e), arguing that acidic pH only affects a portion (likely proportional to time) of DO.

**Figure 4:**
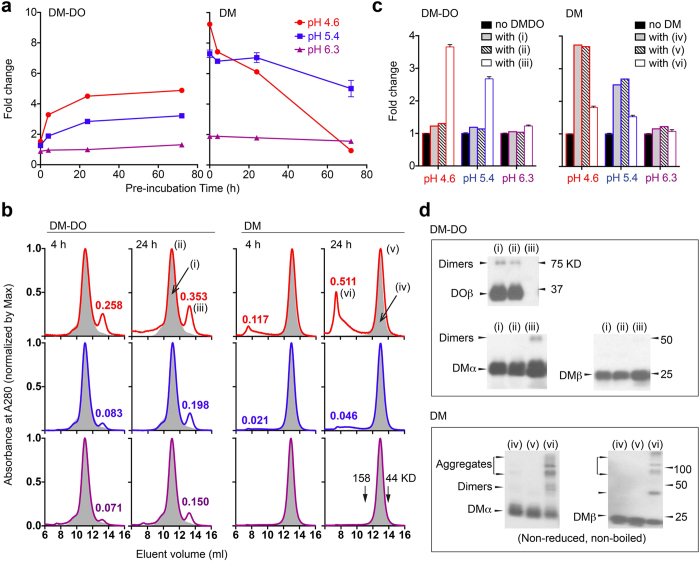
pH-promoted generation of free DM from DM-DO ectodomains. (**a**) DM-DO_wt_ or DM (800 nM) was pre-incubated at the indicated pH at 37 °C and then mixed with DR4 (5 nM) and HA_306–318_ (1 μM). HA loading at the corresponding pH was carried out at 37 °C for 1.5 h. The difference in HA loading with or without acid pre-treated DM-DO (left) or DM (right) was quantified as fold change and plotted against pre-incubation time. (**b**) DM-DO_wt_ or DM was incubated at pH 4.6 (upper panels), 5.4 (middle panels), or 6.3 (lower panels) for indicated times, and the resultant proteins were separated by size exclusion chromatography. The absorbance against eluent volume was normalized. Gray area (i) and (iv) represents eluents of non-treated DM-DO_wt_ and DM, respectively. Relative absorbance of peaks of both novel eluents (iii) and (vi) observed following pre-incubation is indicated. (**c**) Non-treated DM-DO_w_ (i) or DM (iv) and resultant proteins (ii) and (iii) or (v) and (vi) from pH 4.6 pre-treated DM-DO_wt_ or DM were collected separately and used at the same concentration (100 nM DM equivalent) in the HA loading assay described in (**a**). Normalization was performed with peptide loading at the corresponding pH conditions without DM and results are indicated as fold change. (**d**) Proteins in (i–vi) were reduced and boiled, unless otherwise indicated, before separation by gel electrophoresis and then detected with DO- or DM-specific antibodies by western blot. Dimers may include heterodimers and/or homodimers. Error bars represent SEM for triplicates (**a**,**c**). Data are representative of five (**a**) or three (**b**–**d**) experiments.

Based on the previous results, we hypothesized that acid denaturation of DO in DM-DO complexes generates free DM. To test the hypothesis, we analyzed the function of low pH pre-treated DM-DO complexes (double SEC-purified DM-DO ectodomains) in the immunoassay. Untreated DM-DO complexes (pre-incubation time = 0) had negligible effects on peptide exchange (fold change ~1) during <2 h reaction times at all tested pHs (Fig. 4a, left and Supplementary Fig. 7). This result implies that tight association between DO and DM preserves the inhibitory capacity of DO, and DM-DO complexes that promote peptide exchange^27^ have probably released some free DM. As the pre-incubation time at lower pHs increased, DM-DO gradually acquired the capability to promote peptide exchange (reaction pH 4.6 or 5.4), indicated as increased fold change (Fig. 4a, left). On the other hand, peptide exchange catalyzed by acid pre-treated DM was either significantly decreased at pH 4.6 or unchanged at pH 5.4 (Fig. 4a, right). We also observed a decline in fold change after extended exposure of lower amounts of DM-DO complexes to pH 4.6 (Supplementary Fig. 7), which may reflect DM inactivation, similar to pre-incubation of DM ectodomain alone (Fig. 4a, right). Generation of free DM rather than acid-triggered up-regulation of pre-existing DM activity in DM-DO, appears responsible for the enhanced fold change.

To prove that free DM is generated, we isolated proteins from acid-treated DM-DO or DM alone using SEC and then analyzed the function of individual components in the immunoassay. Besides the eluent at 10–12 ml (ii) containing DM-DO complexes, a second eluent at 12–14 ml (iii) with molecular weight 50–60 KD appeared in acid pre-treated DM-DO (Fig. 4b, left). The novel eluent (iii) catalyzed peptide exchanges in a pH-sensitive way (Fig. 4c, left), similar to free DM (Fig. 4c, right). Western blot analyses (Fig. 4d, top) identified DM, but no DO in this eluent (iii), confirming both the generation of DM and the pH-triggered denaturation/destruction of DO previously in complex with DM. As for acid pre-treated DM, a novel fraction (vi) eluted at 7–10 ml (>150 KD) (Fig. 4b, right), and likely contains aggregates, as suggested by western blot (Fig. 4d, bottom). This eluent (vi) showed significantly lower catalytic capacity than untreated DM (iv) or than the eluent containing DM α/β dimers (v) from acid-treated material (Fig. 4c, right). Longer pre-incubation times (24 h vs 4 h) at lower pH (4.6 vs 5.4 vs 6.3) increased the amounts of both novel eluents (Fig. 4c); however, the *in vitro* accumulation of both novel eluents was slow (hours). The slow *in vitro* conversion of DM-DO to denatured DO and free DM may be beyond the detection limit of SPR or BLI analyses. Additionally, pre-incubation of DM-DO ectodomains with or without DR4 gave equivalent results (Supplementary Fig. 8), implying that conversion of DM-DO is not likely triggered by the competitive binding of MHC-II-peptide complex to DM.

### Acid-exposed DM-DO acquires DM activity in cell lysates

**Figure 5:**
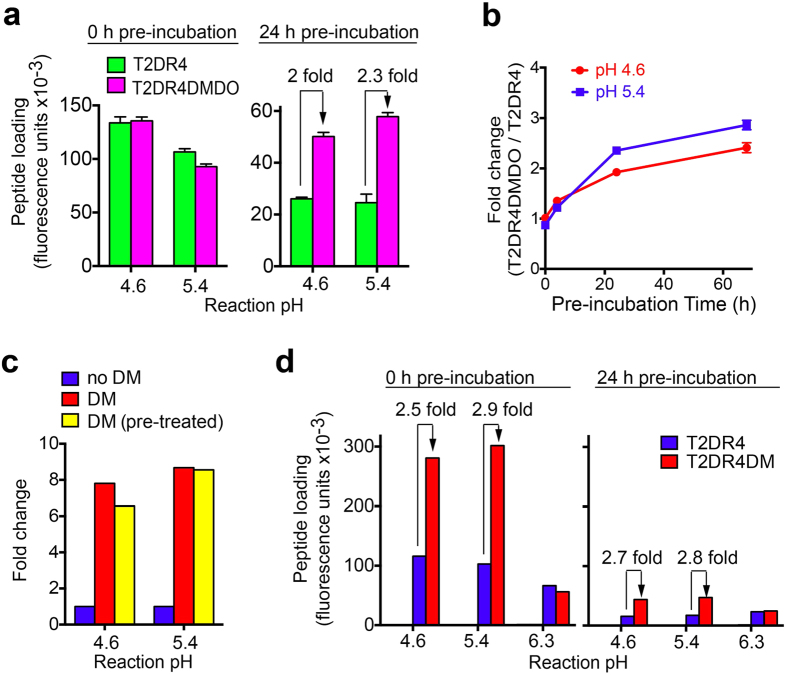
Correlation between acid-exposure of DM-DO and alteration of DM activity. (**a**) Cell lysates of T2DR4 and T2DR4DMDO were pre-incubated at different reaction pHs, 37 °C for the indicated length of time, and then mixed with HA_306–318_ (1.5 μM). HA loading at the corresponding reaction pH was carried out for 1.5 h before measurement of HA bound by full length DR4 quantified as fluorescence units (HA_306–318_ only and lysate only background corrected) and indicated as peptide loading. The fold difference between HA loading in T2DR4DMDO lysates and T2DR4 lysates is indicated. Error bars represent SEM for quadruplicates. (**b**) Fold change in HA loading as measured in (**a**) was plotted against pre-incubation time. (**c**) Native DM purified from T2DM cell lysates was pre-incubated (yellow) at indicated reaction pHs, 37 °C for 24 h or untreated (red) and then mixed with T2DR4 lysate and HA_306–318_ (1.5 μM). HA loading at the corresponding reaction pH was carried out for 1.5 h before measurement of DR4-HA. The fold difference between HA loading with and without DM is shown. (**d**) Peptide loading experiments were performed as in (**a**), but with T2DR4DM lysates and T2DR4 lysates. Background for HA loading, using in T2 or T2DM lysates, was ~zero (not shown). All data are representative of three experiments.

To determine whether full length DM-DO complexes behave similarly to ectodomain complexes, we utilized the immunoassay to compare loading of a hemagglutinin peptide (HA_306–318_) onto full length DR4 in clarified lysates of T2DR4, only expressing one class II allele^28^,^29^; and T2DR4DMDO, expressing DR4 and DM-DO (see **Methods**). This comparison is meaningful, because (1) total DR4 levels in T2DR4DMDO and T2DR4 are comparable, and (2) in both cell lines, CLIP is key component of the DR4 peptide cargo (Supplementary Fig. 9). Without acid pre-treatment, T2DR4DMDO and T2DR4 lysates supported comparable levels of HA loading (1.5 h reaction), indicating that DM function was fully inhibited in the DO/DM expressing cells. Pre-incubation (24 h) of lysates at acidic pH (4.6, 5.4) led to significantly different amounts of HA loading (>2 fold) in the two lysates (Fig. 5a). Indeed, enhanced binding was detectable after 4 hours of pre-incubation and continued to modestly increase after 24 hours, (Fig. 5b). These data strongly suggest that DM activity (promotion of peptide exchange^7^ and/or rescue of empty DR4^4^) results from pre-exposure of full-length DM-DO complexes to acidic pH.

It remained possible that the observed pH effect was due to effects on free DM in the T2DR4DMDO lysate, as pH < 5.5 is known to enhance the activity of soluble recombinant DM in peptide exchange on MHC-II molecules^4^. To address this, we purified full-length DM from T2DM cells and added untreated or acid pre-treated DM to the T2DR4 lysate. Purified DM significantly enhanced HA loading to native DR4 (fold change > 6) at pH 4.6 and 5.4, as expected, but importantly, the fold change (DM activity) was not significantly affected by DM pre-incubation (≥4 h; 24 h) at these acidic pHs (Fig. 5c). In addition, we observed comparable peptide loading of DR4 in T2DR4DM (no DO) and T2DR4 lysates, with or without acid pre-treatment (Fig. 5d). Thus, pH effects on free DM are not likely to explain the effects on T2DR4DMDO lysates described above; the decline in overall HA loading in Fig. 5d is likely due to the acid-promoted denaturation of native DR4 (ref. 4).

## Discussion

Our immunoassays demonstrate that the pH-dependence of DM enzymatic activity is independent of the pH-influence on MHC-II/peptide interaction for the alleles and peptides tested. Given this, optimizing the reaction time frame for peptide exchange, limiting free DM activity, and utilizing a stabilized DO variant to vary DO/DM ratios, especially over 1, allowed us to analyze, and eventually understand mechanistically, the pH effects on DM inhibition by DO.

A key finding of this work is the correlation between the DM concentration and the pH range of its activity. This relationship is reflected in the finding that at suboptimal pH conditions, such as pH > 6.3, we observed DM activity at sufficiently high concentrations of DM. The relationship between DM concentration and activity derives from mass action effects in the one-to-one binding of DM to its MHC II substrate. We previously mapped the DM/DR interface residues and found acidic amino acids at the interface, suggesting an explanation for low DM/DR binding in the ER and increased binding in acidic pH environments^6^,^30^. However, lowering available DM by binding of its competitive inhibitor, DO, may be required as a second, back-up mechanism to block DM/DR interaction in the higher pH, early endocytic compartments in those cells where DM levels are high, but foreign antigen is not present.

We also validated the ability of DO to inhibit DM across a broad pH spectrum (4.5–7.2), as long as DM binding by DO is maintained through DO molar excess. Effective blockade of DM function is lost in the lysosomal pH window, when DO/DM ratios decrease. At a DO/DM ratio <1 (physiological), DM-mediated peptide exchange in lysosomes can proceed.

It has long been speculated that DM-DO complexes may dissociate in lysosomes, but the actual molecular mechanism for pH regulation of DM-DO function has been undefined. To overcome confounding pH influences on individual techniques, we examined the interaction between soluble DM and DO proteins using four independent approaches: ELISA, SPR, BLI, and FRET. Very strikingly, under no circumstance was dissociation of the two heterodimers observed, although we consistently detected fewer DM-DO complexes at lower pH. This appeared mainly due to irreversible, acid-promoted destruction of the DO ectodomain, as also indicated by SEC/western blot results. However, any surviving DO molecules still bound DM tightly even at pH ≤ 4.85, where dissociation was almost undetectable by sensitive SPR analysis. This supports the idea that the molecular interaction between intact DO and intact DM is irreversible, and the separation of DM-DO at acidic pH relies on structural alteration^31^ of the DO ectodomain. The DO structural change may be reversible when DO is still bound by DM as suggested^14^, but is apparently irreversible when it leaves the complex, as we demonstrate here. pH moderately alters DM structure^32^, favoring association with MHC-II^12^ and catalysis of peptide exchange. However, this pH-sensitivity of DM apparently does not drive the acid-dependent DO alteration, which regulates DO/DM ratios at the lysosomal pH.

Importantly, purified DM-DO free from DM contamination allowed us to eliminate another possibility that DM-DO undergoes conformational change to unmask DM function^31^,^32^. Similarly, initial rates of HA peptide binding to DR1 in the presence of DM-DO show no DM effect across the pH 4.5–7.0 range^26^. Taking all of these observations together, we propose a mechanistic model of DM-DO function in which acid-promoted DO destruction results in generation of free DM from DM-DO complexes. However, *in vitro,* a significant percent of DM-DO is resistant to the most acidic lysosomal pH, whereas free DO is very susceptible to pH-induced denaturation. Given these observations, the finding that both DO and DM are detectable in lysosomes^16^,^21^,^22^, suggests they are in complexes. Using the immunoassay along with SEC analysis, we demonstrated the generation of free, functional DM from pre-incubated DM-DO ectodomains. Therefore, free DM rather than DO-bound DM is responsible for the peptide-loading enhancement activity that we observe in soluble DM-DO complexes at acidic pH.

Our immunoassay is sensitive to changes in peptide loading on MHC-II in cell lysates, where all the relevant molecules are full-length. Strikingly, after ≥4 h of pre-incubation at pH 4.6 or 5.4, the lysate from DM-DO expressing cells acquired DM-like activity that either promoted peptide loading or rescued MHC-II. This is the first evidence to suggest possible *in vivo* consequences of acid exposure of DM-DO complexes and is consistent with the mechanism that acidic pH down-regulates DO/DM ratios in favor of free DM. DO levels are known to be reduced in association with activation of B cells and DO-expressing dendritic cells (DCs), influencing the spectrum of antigens that these APC present to CD4+ T cells^10^,^18^,^19^,^20^. Our finding suggests that, in addition to the transcriptional down-regulation of DO expression induced by cell activation stimuli^18^,^20^, the acidification of late-endosomes driven by receptor-activated signaling pathways^33^,^34^ will promote down-regulation of DO/DM ratios (as a result of DO destruction), leading to increased free DM level and activity. Conversely, interference with late-endosomal acidification by chloroquine would be expected to abrogate free DM generation and reduce antigen presentation, especially in high DO-expressing cells^35^.

Notably, the *in vitro* process of DO removal due to decreased pH is slow. However, it would likely be significantly faster *in vivo,* where pH changes would be coupled with activation of acid-active lysosomal proteases. In our immunoassay, a complete set of inhibitors disables proteases, including those in lysates. In addition to proteases, other factors associated with cell activation and signaling, such as redistribution of membrane proteins among internal structures within endosomal compartments^21^,^22^, may also alter the pace and extent of DO denaturation and release of free DM.

Antigens internalized by specific receptors (rather than proteins endocytosed by fluid-phase) are efficiently trafficked to late endosomes^36^ where they are processed and loaded onto MHC-II. For this loading to be efficient, it is likely that MHC-II must pass through early endosomes without peptide editing for tight-binding complexes. DO inhibition of DM may protect against early peptide exchange and editing of MHC-II. Indeed, this may explain why DO knock-out mice, which lack this selectivity for late endosomal sources of antigen, have delayed T-dependent humoral responses to exogenous model antigens and also develop responses to self antigens (e.g., antinuclear antibodies)^37^. Our model that DO acid susceptibility fine-tunes DO/DM ratios suggests a way in which DO, once expressed, may facilitate differential handling of antigens from distinct sources, but still leaves open questions such as why some APCs require DO expression, but others do not, and how DO level is regulated during the differentiation of DO-expressing APCs. For example, germinal center B cells presumably require the broadest pH range of DM activity to maximize antigen presentation, potentially favoring B cell maturation and development^10^,^38^, therefore, the expression of DO as well as the tuning of DO/DM ratios may be unnecessary for cells in the germinal center. However, after B cells exit germinal center, antigen selectivity may be advantageous, and up-regulation of DO expression as well as our proposed mechanistic fine-tuning arises, in for example, memory B cells. Notably, it has been suggested that transcriptional regulation is not responsible for the reduced DO/DM ratios in B cells that enter/are entering germinal center^19^; thus, it is very likely that DO destruction, supported by our model, provides a mechanism.

Interestingly, the ectodomain of DM, unlike full-length DM, had reduced activity at pH 4.6, raising the possibility of an additional role for DO as a stabilizer of the functional domain of DM. Evidence from transgenic mice suggests this effect of DO on DM levels^39^. Chaperoning by DO would end when lysosomal conditions yield free DM, which either catalyzes class II antigen loading or turns over, likely as a consequence of aggregation. This scenario is consistent with evidence that down-regulation of DO during APC maturation/activation is accompanied by a concomitant decline of DM level, though DM/DO ratios increase^10^,^19^,^20^.

Besides ectodomains, the transmembrane domains and cytoplasmic tails of DM-DO complexes may also contribute to acid susceptibility to alter *in vivo* DO/DM ratios. Although a compact form of MHC-II-peptide complex was unable to trigger DM-DO separation using soluble molecules *in vitro*, competition between a transient peptide-receptive form of native MHC-II and DO for binding DM may accelerate DM-DO separation in living cells. Thus, multiple other factors may be additive or even synergistic with pH-dependent tuning of DO/DM ratios.

## Methods

### Cell lines, proteins and peptides

Stable transfectants (Supplementary Table 2) of the TxB hybrid cell line T2 (MHC-II^−^/DM^−^/DO^−^)^40^ or Schneider 2 Drosophila melanogaster insect (S2) cells (Life Technologies) were constructed for protein expression and native or recombinant HLA proteins purified as detailed in Supplementary Information. Biotinylated peptides (synthesized by Genscript) include: HA_306–318_ (influenza hemagglutinin residue 306–318: PKYVKQNTLKLAT), which binds DR^41^; EBV_486–500_ (Epstein-Barr virus nuclear antigen 1 residue 486–500: RALLARSHVERTTDE), which binds DQ6 (ref. 42), MBP_86–99_ (myelin basic protein residue 86–99: NPVVHFFKNIVTPR), which binds DQ1 (ref. 43).

### Immunoassay and kinetic model for peptide loading

Native or recombinant DR or DQ proteins and corresponding biotinylated peptides were mixed in different pH reaction buffers (100 mM buffer strength) containing 0.5% (v/v) IGEPAL CA-630 and protease inhibitors. The peptide exchange reaction in the presence or absence of native or recombinant accessary molecules (DM, DOv, and DM-DO ectodomains) took place at 37 °C for various time length (1–2 h was determined to be optimum to measure pH influences of DM effects). At the end of each reaction, two volumes of ice-cold neutralization buffers were added to stop the reaction. MHC-II dimers loaded with biotinylated peptides were quantified by capture ELISA pre-coated with MHC-II-specific antibodies, with streptavidin-europium detection, as previously described^26^,^44^. Detailed assay methods can be found in Supplementary Information. The *in vitro* peptide loading is a process of peptide exchange with low affinity peptides replaced by high affinity biotinylated peptides. When the dissociation of low affinity peptides are not limiting, the reaction can be approximated as the association between free MHC-II proteins and biotinylated peptides. If the concentration of the biotinylated peptide is significantly higher than the concentration of the MHC-II protein ([*pep*]≫[MHC], or [*pep*]≈constant), the association can be considered as a pseudo-first-order equimolar reaction


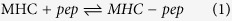


with the reaction rate given by


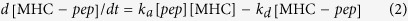


where [MHC*−pep*] represents the concentration of the complex formed at time t; *k*_*a*_ and *k*_*d*_ are the association (on) and dissociation (off) rate constants, respectively. [MHC] can be expressed as [MHC*−pep*]_max_ − [MHC − *pep*] with [MHC − *pep*]_max_ representing the maximum concentration of complexes that can be formed if reaction (1) is irreversible. *k*_*d*_ can also be replaced by *k*_*a*_*K*_*D*_, where *K*_*D*_ represents the equilibrium dissociation constant, which equals *k*_*d*_/*k*_*a*_ as defined. Thus, equation (2) can be rearranged to a first-order linear differential equation


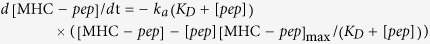


and solved with the initial concentration of complexes [MHC*−pep*]_0_ = 0 to get the general solution


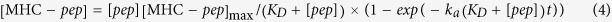


At any given [*pep*] (≈constant), equation (4) can be rewritten as


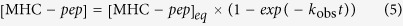


where *k*_*obs*_ is equal to *k*_*a*_(*K*_*D*_ + [*pep*]), representing the observed association rate constant at the given concentration of peptide, and [MHC*−pep*]_*eq*_ is the equilibrium concentration of complexes formed at the given peptide concentration, which is inversely associated with the equilibrium dissociation constant.


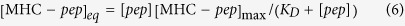


By setting up [*pep*]≫[MHC] (or [pep]≫[MHC−*pep*_low affinity_]), we can measure [MHC–*pep*] at different time points and fit the data to equation (5). In the presence of a catalyst (e.g., DM), both *k*_*a*_ and *k*_*d*_ increase, whereas the *K*_*D*_ stays the same. Thus, the fitted curve using equation (5) should plateau faster as *k*_*obs*_ increases.

### SPR analysis for a slow dissociation kinetics

80 mM citrate-phosphate (disodium phosphate and tri-sodium citrate) running buffers with 150 mM NaCl, 0.06% (w/v) BSA, and 0.05% (v/v) P20 (GE Healthcare) were made at pH 4.85, 5.49, 6.26, and 7.16, respectively. 120–130 resonance units (RU) of biotinylated DM (ligand) were immobilized onto the sensor surface of flow cell 4 (fc4) of a streptavidin sensor chip (GE Healthcare) at a flow rate of 5 μl/min in the running buffer with pH 5.49. SPR data at different pHs were collected at 25 °C or 37 °C on a BIAcore system (GE Healthcare). The system was primed at least 3 times after a switch of running buffers or temperatures. Various concentrations of DO_v_ (analyte) were diluted in the running buffer with the indicated pH and injected using the “Kinject” mode through fc3 (a reference streptavidin sensor) and fc4 at a flow rate of 30 μl/min for 5 min, followed by a dissociation step (running buffer alone for 5–8 min). At the end of each binding cycle, the chip was regenerated by 2–3 quick injections of 50 mM CAPS buffer (pH 10.5) at a flow rate of 100 μl/min for up to 2 min. A single cycle mode with extended dissociation was selected to monitor dissociation of DM-DO_v_ complexes formed on the BIAcore sensor at pH 4.85. The single cycle kinetics included 5 continuous injections of increased concentrations of DO_v_ (7.41, 22.22, 66.67, 200, 600 nM) followed by an extended dissociation over 4 hour. No baseline drifting or mass transfer was observed (not shown). Resonance signal traces acquired for fc3 were subtracted from those obtained for fc4 to reveal the apparent binding kinetics. Immobilization of biotinylated DM at different pHs or blockade of fc3 and fc4 with free biotin showed no effect on DO_v_ binding (not shown). SPR used a sensor surface to immobilize DM and allowed real-time measurement of the interaction between the immobilized DM and DO_v_ in microfluid. When mass transfer and the change in bulk concentration of DO_v_ are negligible, the interaction can be considered as a pseudo-first-order equimolar reaction. Equations (5) and (6) derived above for the study of peptide exchanges can be adapted to the analysis of DO_v_/DM binding kinetics, and rewritten as,


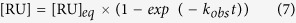


and


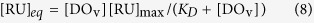


Where [RU]_max_ is determined by the amount of immobilized DM, [RU]_*eq*_ is the equilibrium concentration of complexes formed at the give concentration of DO_v_. Equation (6) is equivalent to the Langmuir equation for the single-site binding model. Notably, the advantage of using equations (7) and (8) to analyze SPR data is that the dissociation curve is not necessary for the data fitting, but we have to make the assumption that the interaction between DO_v_ and DM fits the slow dissociation kinetics.

### Bio-layer interferometric analysis

A set (up to 8) of streptavidin biosensors (Fortebio) were hydrated in a reaction buffer for at least 1 h, and then positioned into an Octet QK system (Fortebio). The automatic system allows biosensors to dip into a column of wells in a black 96-well plate (E&K Scientific) containing biotinylated DO_v_ (ligand) or soluble DM (analyte) sequentially. To minimize mass transfer and the depletion of analyte at the analyte association step, the biosensors were only incubated with biotinylated DO_v_ for 2–5 min, allowing a trace amount of DO_v_ immobilization (1.7 nm wavelength shift), and then washed with the kinetic buffer for 5–10 min before dipping into DM-containing wells. After association, the dissociation of DM-DO_v_ complexes formed on the biosensors was analyzed by dipping biosensors into 200 μl kinetic buffers for up to 2 hours. To monitor the dissociation of wild type DM-DO complexes in a separate experiment, the streptavidin biosensor was first immobilized with biotinylated DM-DO_wt_ and then immerged in wells with 200 μl kinetic buffers for over 4 fours. Assays were performed at 25 °C or 37 °C with the plate shaking at 1,000 rpm. 24 nM biotinylated DO_v_ or 100 nM biotinylated DM-DO or various concentrations of soluble DM were prepared in 200 μl of kinetic buffers at the indicated pHs in one assay. Kinetic buffers containing 150 mM NaCl, 0.01% (w/v) BSA, 0.002% (v/v) Tween 20 were made in 50 mM one of the following buffers: acetate buffer, pH 4.7; citrate buffer, pH 5.5; MES buffer, pH 6.3; and HEPES buffer, pH 7.4. No binding of irrelevant proteins to biotinylated DO_v_ was observed (not shown). Data were background corrected.

### FRET analysis

The free cysteine on the surface of DM(βC46), DOvβS63C, and DOvαR80C was reduce by DTT and crosslinked to the reactive CyDye maleimides (GE Healthcare). DM-Cy5, DOvβS63C-Cy3 and DOvαR80C-Cy3 were separated from free CyDye maleimides by SEC using a Superdex 200 column (GE Healthcare) and mixed in a pH buffer before acquisition of the emission spectrum (560–700 nm) using a Gemini XS fluorescent microplate reader (Molecular Devices). The activity of CyDye labeled proteins was verified using peptide-loading assays.

### Western blotting analysis

Non-treated proteins or proteins reduced by β-mercaptoethanol followed by boiling at 100 °C for 10 min were separated by gel electrophoresis using 12% precast polyacrylamide gels (Bio-Rad). Proteins were then transferred to Immobilon-P membrane (Millipore) for antibody labeling and detection.

## Additional Information

**How to cite this article**: Jiang, W. *et al.* pH-susceptibility of HLA-DO tunes DO/DM ratios to regulate HLA-DM catalytic activity. *Sci. Rep.* **5**, 17333; doi: 10.1038/srep17333 (2015).

## Supplementary Material

Supplementary Information
