# Supplementary material for: pH-susceptibility of HLA-DO tunes DO/DM ratios to regulate HLA-DM catalytic activity
Source: Sci Rep. 2015 Nov 27;5:17333. doi: 10.1038/srep17333 (PMC4661524; doi:10.1038/srep17333)
Supplement: Supplementary Information [file srep17333-s1.pdf]

Supplementary Information

pH-susceptibility of HLA-DO tunes DO/DM ratios to regulate HLA-DM catalytic activity

Wei Jiang, Michael Strohman, Sriram Somasundaram, Sashi Ayyangar,

Tieying Hou, Nan Wang, and Elizabeth D. Mellins

Supplementary Information includes

- **Supplementary Methods**
- **9 Supplementary Figures**
- **4 Supplementary Tables**
- **References**

Supplementary Methods

Cell lines

Stable transfectants (**Supplementary Table 2**) of the TxB hybrid cell line T2 (MHC-II$^-$/DM$^-$/DO$^-$)[1] were cultured in complete Iscove's Modified Dulbecco's Medium (IMDM with GlutaMax and 10% heat inactivated FBS, Life Technologies) to express native class II proteins. The expression of target proteins in T2 transfectants was selected by specific antibiotics (**Supplementary Table 2**) for at least 1 week prior to cell lysis or analysis.

Stable transfectants (**Supplementary Table 2**) of Schneider 2 Drosophila melanogaster insect (S2) cells (Life Technologies) were maintained and induced in serum-free insect cell medium (BD Biosciences) at room temperature (RT) to express soluble recombinant ectodomains of class II proteins. 1 mM CuSO4 was used to induce protein expression at a cell density of 10-20 million cells/ml for at least 7 days before harvesting the cells for protein purification.

Construction of plasmids for protein expression in transfected cell lines

DOA*0101 and DOB*0101 encoding α and β chains of wild type DO, respectively, were amplified from pBMN-based DO-expressing plasmids[2] by standard PCR using template-specific primers (see **Supplementary Table 3** for all primers used in plasmid construction). The two amplified PCR products (amplicons) were then sequentially subcloned into pBudCE4.1 (zeocin-resistant, Life Technologies) via two pairs of unique restriction enzyme (RE) recognition sites (NotI/BglII for DOB amplicon and SalI/BamHI for DOA amplicon) within the two multiple cloning sites. The resulting plasmid pBudCE4.1-DOA/DOB, directs simultaneous expression of DOα and DOβ, under two independent human promoters in transfected mammalian cell lines.

To construct plasmids directing the expression of DQ6, DQA1*01:02 encoding DQ6 α chain was amplified by PCR using complementary DNA (cDNA) made from PBMCs of an HLA-DQA1*01:02 homozygote donor. DQB1*06:02 encoding DQ6 β chain was amplified by PCR from the bicistronic pLNCXPOX vector containing the DQ6B cDNA (a gift from Dr. William Kwok, Benaroya Research Institute, Seattle, WA). DQ6A and DQ6B amplicons were subcloned into the retroviral vector PBMN-ZIN-neo via BamHI/SalI and EcoRI/SalI RE recognition sites to construct plasmids: pBMN-ZIN-neo-DQ6A and pBMN-ZIN-neo-DQ6B, respectively.

As for plasmids directing the expression of recombinant, soluble DQ6 in complex with CLIP peptide in S2 cells, the regions in DQA1*01:02 and DQB1*06:02 encoding the ectodomains of α and β chains of DQ6, respectively, were amplified from vector pMT-DQA1*0102 and pMT-DQB1*0602-OR1-13 (gifts from Dr. Lars Fugger, Weatherall Institue of Molecular Medicine, UK) and subcloned into separate pRmHa3 vectors via EcoRI/SalI and BspEI/SalI RE recognition sites, respectively. The sense primer for sDQ6B amplicon was designed such that $CLIP_{87-101}$ (residue 87-101:PVSKMRMATPLLMQA) was covalently tethered to the N-terminus of β chain via a flexible linker bearing the thrombin cleavage site (LVPRGS). Antisense primers for sDQ6A and sDQ6B carried an extra unique RE recognition site (SacI or KpnI) for a second step PCR to introduce C-terminal leucine zipper -Fos and -Jun segments in pRmHa3-sDQ6A-Fos and pRmHa3-sDQ6B-Jun, respectively.

Similar constructs were used in plasmids directing the expression of soluble DQ1 in complex with CLIP in S2. The regions in DQA1*01:01 and DQB1*05:01 encoding the ectodomains of α and β chains of DQ1, respectively were amplified by PCR using cDNAs made from a B lymphoblastoid cell line 3.1.3. The two amplicons were subcloned into plasmids pRmHa3-sDQ2A and pRmHa3-sDQ2B, respectively, directing expression of soluble DQ2 (ref [3]), in place of the DQ2A and DQ2B genes. The resulting plasmids are pRmHa3-sDQ1A and pRmHa3-sDQ1B, respectively. The latter allowed the expression of a flag-tag (DYKDDDDK) at the C-terminal end of DQ1 β chain for purification and detection purposes.

Generation of stable cell transfectants

T2DR4DM was transfected with pBudCE4.1-DOA/DOB by nucleofection, using Amaxa nucleofector kit C (Lonza) specific for T2 cell lines to construct a stable DO-expressing cell line. The transfectants were cultured in complete IMDM medium with 50-100 μg/ml zeocin (Life Technologies) for 3-5 weeks, and then sorted for surface CLIP$^+$ cell populations by fluorescence-activated cell sorting (FACS). Over 99% of the sorted cells, T2DR4DMDO, expressed DO (**Supplementary Fig. 9**). pBMN-DQ6A and pBMN-DQ6B containing DQA1*01:02 and DQB1*06:02 cDNA, respectively, were sequentially introduced into T2 cells, using the Phoenix Retroviral System, as described[3]. Cells expressing DQ6 dimers on the surface were enriched using phycoerythrin (PE)–conjugated anti-DQ antibody (Ia3 clone, BD Biosciences) and anti-PE MACS microbeads (Miltenyi Biotec) to generate cell line T2DQ6. Expression of DQ6 (>99%) on the surface of cells was confirmed by flow cytometry.

S2 cells were co-transfected following the standard protocol (Life Technologies) with three plasmids directing the expression of recombinant DQ α chain , β chain, and the neomycin (geneticin)-resistance gene, respectively, at a ratio of 20:20:1. The transfected S2 cells were selectively cultured in complete Schneider Drosophila medium (with 10% FBS and 2mM Glutamine, Life Technologies) containing 1.5 mg/ml Geneticin (G418, Life Technologies) for 4-5 weeks to construct a stable cell line secreting recombinant DQ6 or DQ1 proteins.

Immunofluorescent labeling and flow cytometric analysis

Transfected T2 cells were fixed and permeabilized using the Cytofix/Cytoperm kit (BD Pharmingen). Washed cells were resuspended in monoclonal antibody (mAb) containing solution at a density of 1 million cells per 100 μl and stained on ice with fluorophore-conjugated mAbs. These mAb include, FITC-CerCLIP (Fluorescein isothiocyanate-conjugated anti-human CLIP mAb, used at 1:5 dilution, BD Pharmingen), PE (R-phycoeryhthrin)-conjugated anti-HLA-DR mAb (used at 1:5 dilution, BD Pharmingen), Alexa fluor 568-MagsDO5 (anti-human DO mAb[4], used at 1:50 dilution), Alexa fluor 647-MapDM1 (anti-DM mAb[5], used at 1:200 dilution). To label proteins (e.g., CLIP, DR and DQ) expressed on cell surface, mAb staining was performed without fixation and permeabilization. Labeled cells were washed twice with generous amount of Permwash buffer (for permeabilized cells, BD Pharmingen) or PBS+1% BSA (for surface-stained cells) and resuspended in PBS+1% BSA before the analysis on a flow cytometer or cell sorting using a FACSAria cell sorter (Becton Dickinson). Flow cytometric data were analyzed using FlowJo software (Tree Star, Inc.).

To make Alexa fluor dye conjugated mAbs, MagsDO5 and MapDM1 were first purified from the culture supernatant of mouse hybridoma (a gift from Dr. Lisa Denzin, Rutgers, the State University of New Jersey) and ascites, respectively, using protein G beads (GE Healthcare), and then labeled with corresponding Alexa Fluor dye carboxylic acid, succinimidyl ester (Life Technologies), followed by size exclusion chromatography using a Superdex 200 gel filtration column to remove extra free dye.

Cell lysis and purification of full length DM

Stable T2 trasfectants were lysed at a density of 0.2 million cells/μl in tris buffered saline (TBS, 20 mM Tris-Cl, 150 mM NaCl, pH 7.4) + 1% (v/v) IGEPAL CA-630 (Sigma) or 1% (w/v) CHAPS (MP Biomedicals, LLC) + 1.5X EDTA-free complete protease inhibitor (Santa Cruz Biotechnology, Inc.) with vigorous shaking at 4°C overnight. Clarified cell lysates were spun and filtered through ultrafiltration spin-columns (0.22 or 0.45 μm, Millipore). Proteins in the lysate were affinity-purified or used directly in the immunoassay for peptide loading.

MapDM1 mAbs were coupled with CNBr-activated sepharose 4B (GE Healthcare) before packing into a column for DM purification. DM was purified from the clarified T2DM lysate (from 500 million cells) by affinity chromatography using the customized MapDM1 column and eluted from the column using an acidic buffer (100 mM glycine-HCl, 150 mM NaCl, pH 3) + 0.5% (w/v) CHAPS. The eluent was immediately neutralized with 1M Tris-Cl (pH 8.5) + 0.5% (w/v) CHAPS and analyzed by western blot and size exclusion chromatography.

Purification of recombinant proteins

The supernatant of S2 cell culture containing soluble proteins were filtered through a 0.22 um membrane to remove cell debris, and proteins were purified from the supernatant by affinity chromatography, followed by size exclusion chromatography, using a Superdex 200 gel filtration column (GE Healthcare). Fractions containing monomeric forms of each protein were pooled and analyzed by Coomassie staining or western blotting. For affinity chromatography, customized L243 (DR-specific mAb) columns[6] were used to purify DR1, DR3, and DR4; a customized SPV-L3 (DQ-specific mAb)[7] column was used to purify DQ6; and M2 (a flag-tag-specific mAb) columns (Sigma) were used to purify DQ1, DM, DO_v, DO mutants and wild type DM-DO complexes, which have flag-tag epitopes covalently attached at the C-terminus of one chain. A low buffer-strength running buffer (e.g., 20-25 mM Tris-Cl, 150 mM NaCl, pH 7.4) typically was used for size exclusion chromatography to minimize the dilution/neutralization effect on the downstream pH-sensitive assays. The SPV-L3 mAb was purified from the culture supernatant of mouse hybridoma using gammabind G sepharoses (GE Healthcare) and then coupled with CNBr-activated sepharose 4B (GE Healthcare) before packing into a column. Some soluble DR4 with covalently tethered CLIP was kindly provided by Drs. Laura Su and Mark Davis (Stanford). Ectodomains of wild type DM-DO complexes were gel filtration-purified twice to eliminate any free DM.

Protein biotinylation and thrombin cleavage

An avitag (GLNDIFEAQKIEWHE) was added to the C-terminus of DM, DO_v, and wild type DM-DO ectodomains, respectively, allowing site-specific biotylation using

BirA ligase (Avidity). The biotinylation reaction was performed in the presence of BirA enzyme at RT for 1 h and then kept at 4°C overnight before the removal of excess free biotins by repeated concentration and buffer-exchange using an Amicon Ultra-15 (10 KDa MW cut off, Millipore). The resultant proteins are designated as bio-DM, bio-DO_v, and bio-DM-DO, respectively. Recombinant MHC-II allelic proteins covalently tethered with $CLIP_{87\text{-}101}$ were treated with thrombin (at 1 U/100 μg protein, Novagen) for 2 h at RT to cut the linker between the CLIP peptide and the MHC-II α/β dimer.

Immunoassay for quantification of DM effect at varied pH

A series of high strength (100 mM) buffers were made for the peptide exchange reaction, and pH was monitored before and after the introduction of reaction components to ensure precise pH. These reaction buffers contain 150 mM NaCl, 1% (w/v) BSA, 0.5% (v/v) IGEPAL CA-630 (Sigma), 0.1% (w/v) NaN3, and 100 mM of one of the following buffers: acetate buffer (acetic acid and sodium acetate), pH 4.6; citrate buffer (citric acid and sodium citrate), pH from 4.56 to 5.41; phosphate buffer (monosodium phosphate and disodium phosphate), pH 6.3 or 7.2; (see **Supplementary Table 4** for a comparison of pH measured at different temperatures). 1X EDTA-free complete protease inhibitor (Santa Cruz Biotechnology, Inc.) was added to each reaction buffer before use. Separate neutralization buffers, all consisting of 150 mM NaCl, 1% (w/v) BSA, 0.5% (v/v) IGEPAL CA-630, 0.1% (w/v) NaN_3, and 100 mM of an appropriate pH buffer, were made for reactions at different pHs (e.g., neutralization buffer in 100 mM Tris-Cl, pH 8.4 can be used for reactions at pH 4.6 to pH 4.9).

In the time course experiment, bulk reaction mixtures containing recombinant MHC-II allelic proteins or native MHC-II in clarified cell lystaes and various concentrations of peptides with or without DM were prepared in reaction buffers at different pHs and incubated at 37°C. Affinity purified soluble and native DM were used to catalyze peptide loading onto recombinant and native MHC-II, respectively. At different time points, a fraction of each reaction was collected and stopped by adding two volumes of corresponding ice-cold neutralization buffer. MHC-II α/β dimers loaded with biotinylated peptides were quantified by capture ELISA with streptavidin-europium detection, as previously described[3, 8, 9]. For the capture ELISA, L243 and SPV-L3 mAbs from ascites were coupled to the high adsorption 96-well plate (Thermo Scientific) for capture of DR and DQ proteins, respectively.

In vitro peptide loading with pre-treated DM and DO

Recombinant accessary molecules, including DM, DO_v, and wild type DM-DO complexes, were pre-diluted into the reaction buffer (described above) or buffer-exchanged to a low buffer-strength pH buffer (see below) with 1X EDTA-free complete protease inhibitor and incubated at 4°C or 37°C for an indicated time before being mixed with the MHC-II protein and the biotinylated peptide. These low strength pH buffers include 150 mM NaCl and 10 mM of one of the following buffers: acetate buffer, pH 4.8; citrate buffer, pH from 4.6 to 5.4; MES buffer, pH 6.3; PBS, pH 7.4. The peptide exchange reaction in the presence of different combinations of accessary molecules (pre-treated versus non-treated) took place at 37°C for 1-3 h as indicated. After incubation, reactions were neutralized and MHC-II loaded with biotinylated peptides were quantified as described above by capture ELISA.

In case of pre-incubation of regulatory molecules with DR4, 2x concentrated mixtures containing 10 nM of recombinant DR4 and various concentrations of soluble DM or DM-DO_{wt} were prepared in reaction buffers (with protease inhibitor) at varied pH and pre-incubated at 37°C. When full-length proteins were utilized, clarified lysates of 0.2 million T2 cells or T2 MHC-II-transfectants were diluted in reaction buffers (with protease inhibitor) at different pHs and pre-incubated at 37°C. After pre-incubation, bio-$HA_{306-318}$ prepared in reaction buffers (with protease inhibitor) with the same pH was added to a final concentration of 1.5 μM and peptide exchange reactions were then took place at 37°C for 1.5 h before neutralization and quantification of HA in association with DR4, as described above. Samples were all prepared and mixed on ice in order to maintain the stability of proteins and to minimize the variation in incubation time at higher temperature. Neutralized reactions were frozen at -20°C before analysis by the capture ELISA if necessary.

Capture of DM-DO by ELISA

200 nM of bio-DM and various concentrations of DO_v were mixed in reaction buffers at different pHs (detailed above) and incubated at 37°C for 1.5 h. After neutralization, the reaction mixture was transferred to a 96-well plate pre-coated with Mags.DO5 mAb for capture of DO_v or DM-DO_v at RT for 1 h. The plate was then washed and the captured complexes were detected by europium-labeled streptavidin (PerkinElmer), using time-resolved fluorescence (Wallac).

Fluorescent dye labeling and FRET analysis

DM, DO_vβS63C, and DO_vαR80C were buffer-exchanged to TBS buffer (50 mM Tris-Cl buffer, 150 mM NaCl, 5 mM EDTA, pH 7.4) using Amicon Ultra-15 (10 KDa MW cut off) and mixed with freshly dissolved DTT to a final concentration of 0.3-0.4 mg protein/ml to reduce the single free surface cysteine for conjugation of CyDye maleimides (GE Healthcare). DM was incubated with 45 mM DTT at RT and then left overnight at 4°C, while DO mutants were incubated with 20 mM DTT plus 1X complete protease inhibitor at RT for 45 min to maintain the stability of DO molecules. Reduced DM and DO mutants were repeatedly diluted in TBS and concentrated using Amicon Ultra-15 (10 KDa MW cut off) to remove excess DTT and then incubated with a 20-40 fold molar excess of Cy5 maleimide and Cy3 maleimide (GE Healthcare), respectively, at RT for 2-3 h and left overnight at 4°C, protected from light. After incubation, the reaction was quenched with 1 mM DTT. Cy5 labeled DM (DM-Cy5) or Cy3 labeled DO mutants (DO_vβS63C-Cy3 and DO_vαR80C-Cy3) were separated from free CyDye maleimides by size exclusion chromatography, using a Superdex 200 gel filtration column. The activity of CyDye labeled proteins was verified using peptide-loading assays (**Supplementary Fig. 5**). CyDye/protein ratios for DM and DO mutants were 0.5 and 0.8, respectively. An irrelevant protein (MHC-I) that contains a free cysteine was also labeled with Cy5 similarly; and the CyDye/protein ratio was 0.5. MHC-I was kindly provided by Dr. Larry Stern (UMass Medical School). 100 μl of 50 mM phosphate buffer, 150 mM NaCl, pH 7.0 or 50 mM acetate buffer, 150 mM NaCl, pH 4.7, containing various concentrations of Cy3, Cy5, DM-Cy5, or MHC-I-Cy5, or DO_vβS63C-Cy3, or DO_vαR80C-Cy3 alone, or any combinations of donor (Cy3) / acceptor (Cy5) pairs were prepared in a black 96-well flat-bottom Costar assay plate (Corning Inc.). Samples were excited by laser at a

wavelength of 500 nm and the emission spectrum in between 560 nm and 700 nm was acquired at 25°C using a Gemini XS fluorescent microplate reader (Molecular Devices), equipped with a 550 nm long-pass filter. Emission values measured for acceptor alone samples were subtracted from those measured for corresponding donor/acceptor samples to calculate the FRET-associated fluorescence signal.

Western blotting analysis

Non-treated proteins or proteins reduced by β-mercaptoethanol followed by boiling at 100°C for 10 min were separated by gel electrophoresis using 12% precast polyacrylamide gels (Bio-Rad). Proteins were then transferred to Immobilon-P membrane (Millipore) for antibody detection. TAL 18.1 (0.2 mg/ml, DMα-specific mAb, Santa Cruz Biotechnology, Inc; used at 1:600 dilution) and 27E8 (His-tag-specific mAb; used at 1:1000 dilution) followed by HRP-goat anti-mouse IgG1 Ab (1mg/ml, Invitrogen; used at 1:10,000 dilution) were used to detect DMα and His-tag at the C-terminus of recombinant DQα, respectively. Rabbit anti-KT3-tag Ab (1mg/ml, Genscript; used at 1:4000 dilution) and rabbit anti-Avitag Ab (0.5 mg/ml, GenScript, used at 1:2000 dilution) followed by HRP-donkey anti-rabbit IgG Ab (GE healthcare; used at 1:20,000 dilution) were used to detect KT3-tag at the C-terminus of recombinant DMβ and Avitag at the C-terminus of recombinant DOβ, respectively. KT3 and Avitag are both unique in DM-DO_{wt}. HRP-anti-flag-tag mAb (1 mg/ml, Sigma; used at 1:10,000 dilution) was used to detect flag-tag at the C-terminus of recombinant DQβ. Sufficient stripping of pre-bound antibodies was performed using western blot stripping buffer (Restore;Thermo Scientific) when blotting of the same membrane was needed.

Supplementary figures

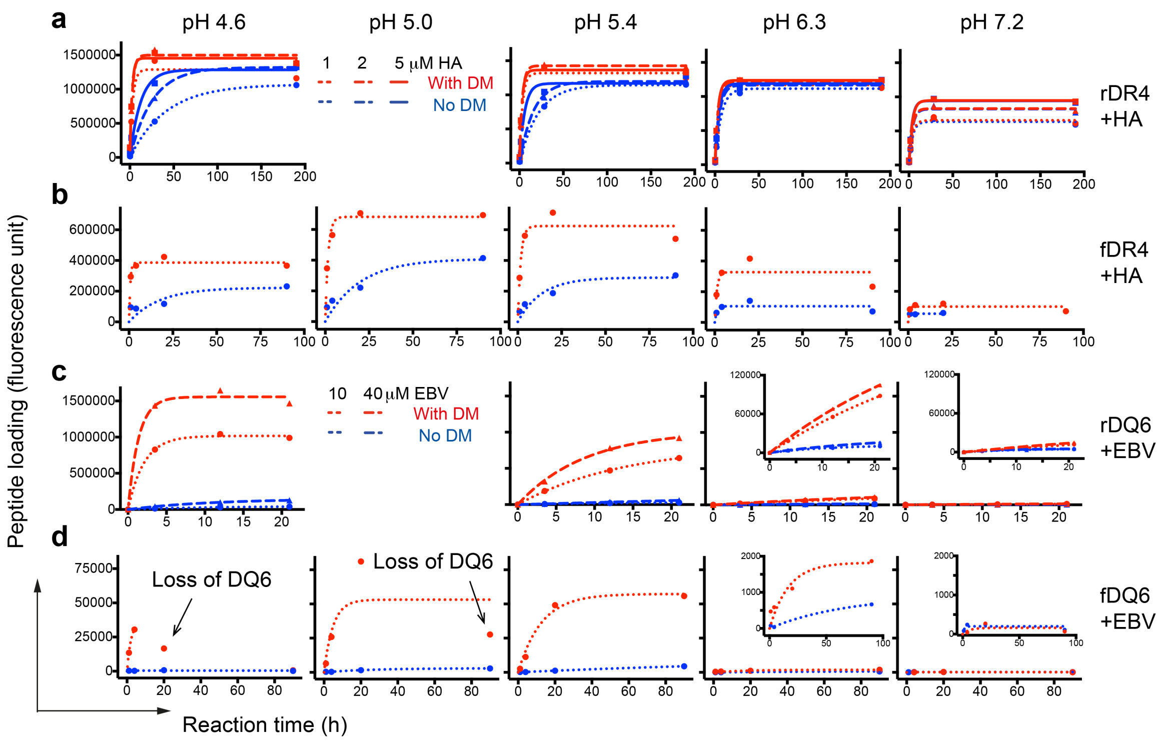

Supplementary Figure 1. Peptide loading as a function of pH.

This figure shows all the raw data and fitted curves for the conversion to fold change in Figure 1c. (**a**) Recombinant soluble DR4 (5 nM) was incubated with indicated concentrations of $HA_{306-318}$ in the presence or absence of soluble DM (200 nM) at the indicated pH for different time, followed by measurement of DR4-HA complexes quantified as fluorescence units ($HA_{306-318}$ only background corrected) and indicated as peptide loading. Data for 1 μM (dot), 2 μM (triangle), and 5 μM (square) of $HA_{306-318}$, respectively, were fit to equation ⑤ $[\text{MHC-}pep] = [\text{MHC-}pep]_{eq} \times (1- \exp(-k_{obs}\, t))$ (see **Methods**). (**b**) Full length DR4 in clarified T2DR4 cell lysates was incubated with 1 μM $HA_{306-318}$ in the presence or absence of affinity-purified native DM at the indicated condition and analyzed as in (**a**). (**c**) Reactions between recombinant soluble DQ6 (40 nM) and the indicated concentrations of $EBV_{486-500}$ were carried out and analyzed similarly to (**a**). (**d**) Reactions between full length DQ6 in clarified T2DQ6 cell lysates and $EBV_{486-500}$ (2 μM) were carried out and analyzed similarly to (**b**). Data represent the mean of duplicates. Some plots were rescaled and shown in the inserted graphs. Arrows indicate significant loss of MHC-II proteins at low pH. Shorter incubation times (<0.5 h) were not practical for parallel reactions, given the set-up time needed for these reactions.

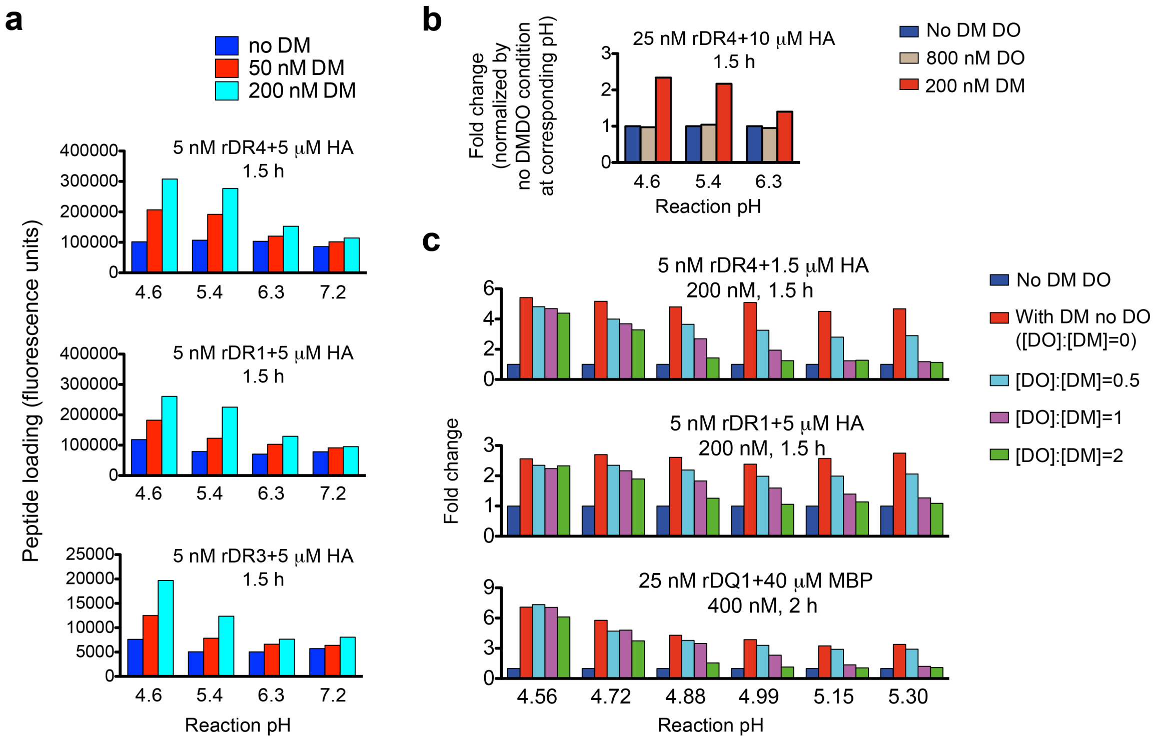

Supplementary Figure 2. pH dependence of DM or DO effects on peptide loading.

This figure shows the raw data for the conversion to fold change in Figure 1d and DM efficiency in Figure 1e. (**a**) Recombinant DR allelic proteins (5 μM) were incubated with $HA_{306\text{-}318}$ (5 μM) with indicated concentrations of soluble DM for 1.5 h, followed by measurement of DR4-HA complexes quantified as fluorescence units and indicated as peptide loading. pH influence on spontaneous peptide exchange did not drive pH effects on DM catalyzed peptide exchange. (**b**) DO has negligible effect on peptide loading. HA loading to DR4 was carried out at the indicated condition with DM or DO, normalized to the condition without DM and DO, and shown as fold change. (**c**) Reactions between different MHC-II proteins and corresponding biotinylated peptides as indicated were carried out with different concentrations of DO_v and DM, normalized to reactions without DM at the corresponding reaction pH, and shown as fold change. Data represent the mean of duplicates.

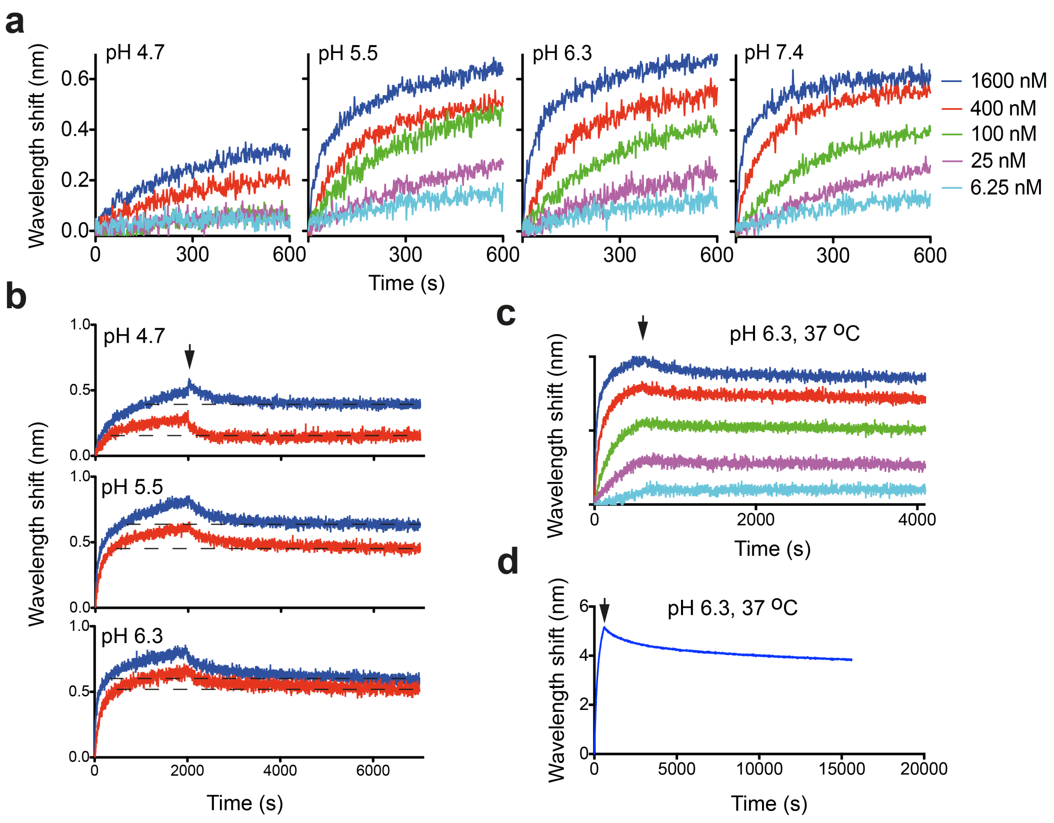

Supplementary Figure 3. Negligible dissociation of DM-DO ectodomains is detected by BLI.

(**a**) Different concentrations of DM, as denoted by colors, were incubated with DO_v immobilized on a sensor tip of the Octet OK BLI system at the indicated pH, 37°C (see **Methods**). The association signal was recorded as wavelength shift and plotted against time. (**b**) After incubation of immobilized DO_v with different concentrations of DM (as in **a**), the sensor was emerged in a well with the indicated pH buffer for detection of the dissociation. Arrows indicate the beginning of dissociation. (**c**) Experiments (as in **b**) were performed at 37°C, pH 6.3. (**d**) Dissociation of wild type DM-DO complexes analyzed by Octet OK at the indicated condition. Temperature effects on the biolayer interface of Octet sensors also contribute to wavelength shifts, but have no influence on measurements of dissociation. Data are representative of three (**a, b**) or two (**c, d**) experiments.

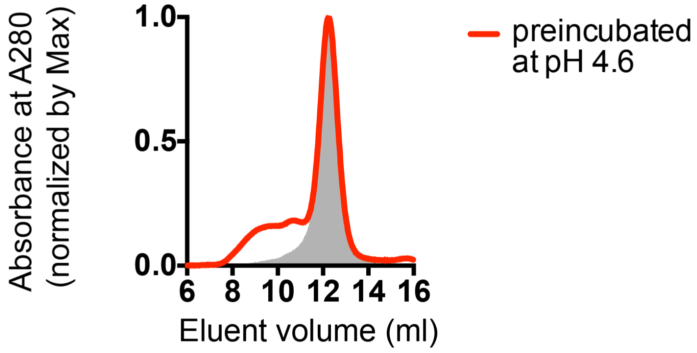

Supplementary Figure 4. Purification of intact DO_v after acidic pH pulse.

Eluents at 12-13 ml, expected to contain intact DO_v based on their elution at the volumes where non-treated DO_v (gray area) elute, were pooled and used for SPR analysis in Figure 2f and peptide loading studies in Figure 3c. To prepare acid-treated $DO_{v,}$ it was incubated at pH 4.6, 37°C for 8 min and separated by size exclusion chromatography using a Superdex 200 gel filtration column. The normalized UV absorbance of pulsed DO_v (red line) is plotted against eluent volume with an overlay of the UV trace for non-treated DO_v.

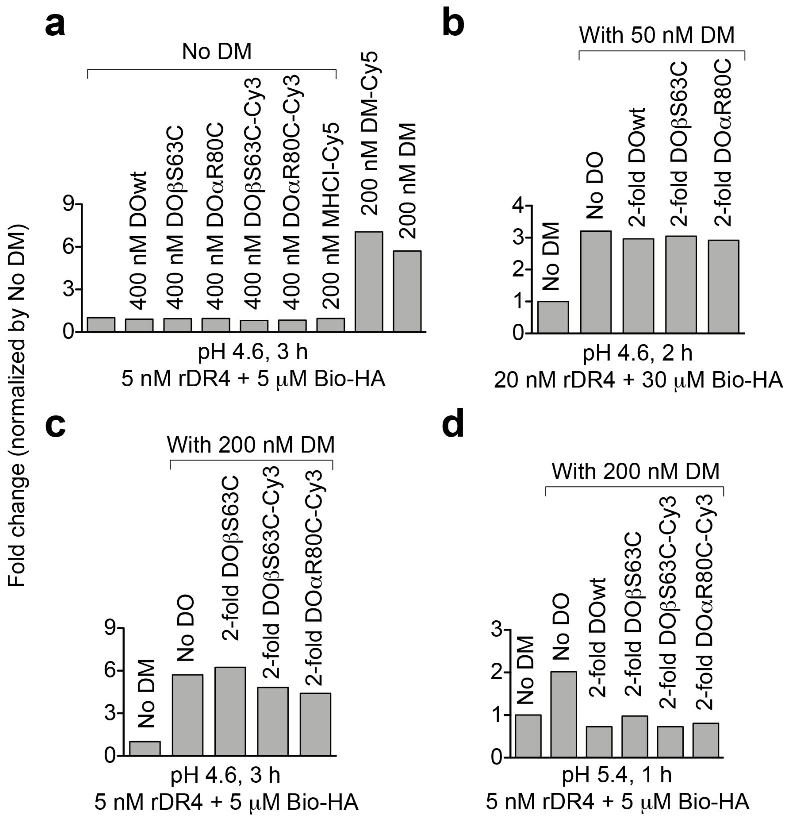

Supplementary Figure 5. Negligible effects on DM or DO$_v$ function after modification for FRET analysis.

DO mutants, Cy3-labeled DO mutants and Cy5-labeled DM all function in a pH-dependent manner, similar to their unmodified counterparts (see **Supplementary Fig. 2** for comparison). Reaction components and conditions are indicated. Data represent the mean of duplicates.

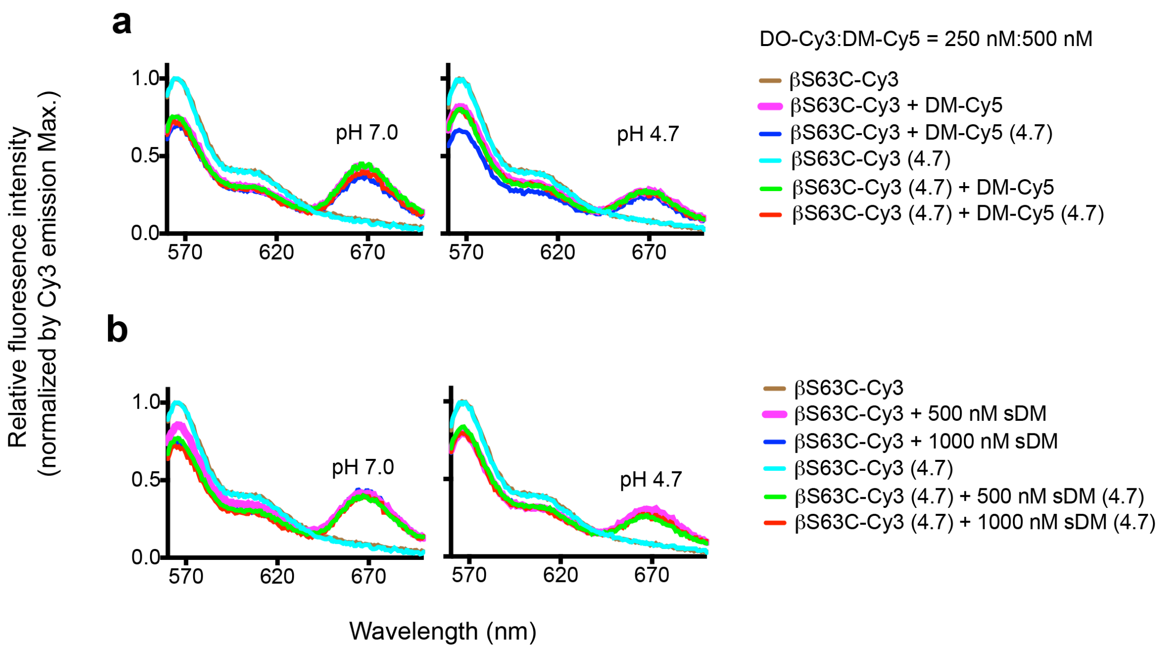

Supplementary Figure 6. Cy3 and Cy5 dyes are not affected by treatment at pH 4.7.

DO_vβS63C-Cy3 or DM-Cy5 was incubated at pH 4.7 as indicated in parenthesis, 37°C for 30 min and then applied to a Superdex 200 gel filtration column to purify intact proteins by size exclusion chromatography. FRET analyses using pH 4.7 pre-treated or non-treated donors (DO_vβS63C-Cy3) and acceptors (DM-Cy5) show no significant differences, indicating that the exposure to pH 4.7 has negligible effects on Cy3 and Cy5 dyes.

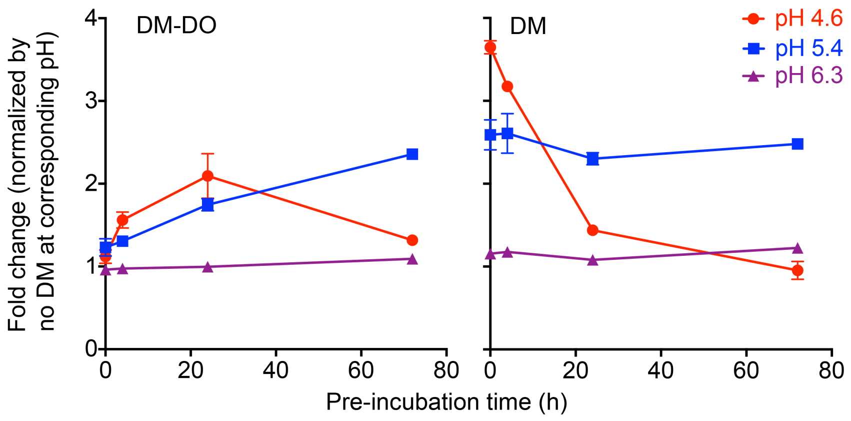

Supplementary Figure 7. Acid-triggered loss of DM activity is detected when lower concentrations of DM-DO are used in the immunoassay shown in Figure 4a.

DM-DO_{wt} or DM (100 nM) was pre-incubated at the indicated pH at 37°C and then mixed with DR4 (5 nM) and $HA_{306-318}$ (1 μM). HA loading at the corresponding pH was carried out at 37°C for 1.5 h. The difference between HA loading with or without DM-DO (left) or DM (right) was quantified as fold change and plotted against pre-incubation time. Error bars represent SEM for triplicates.

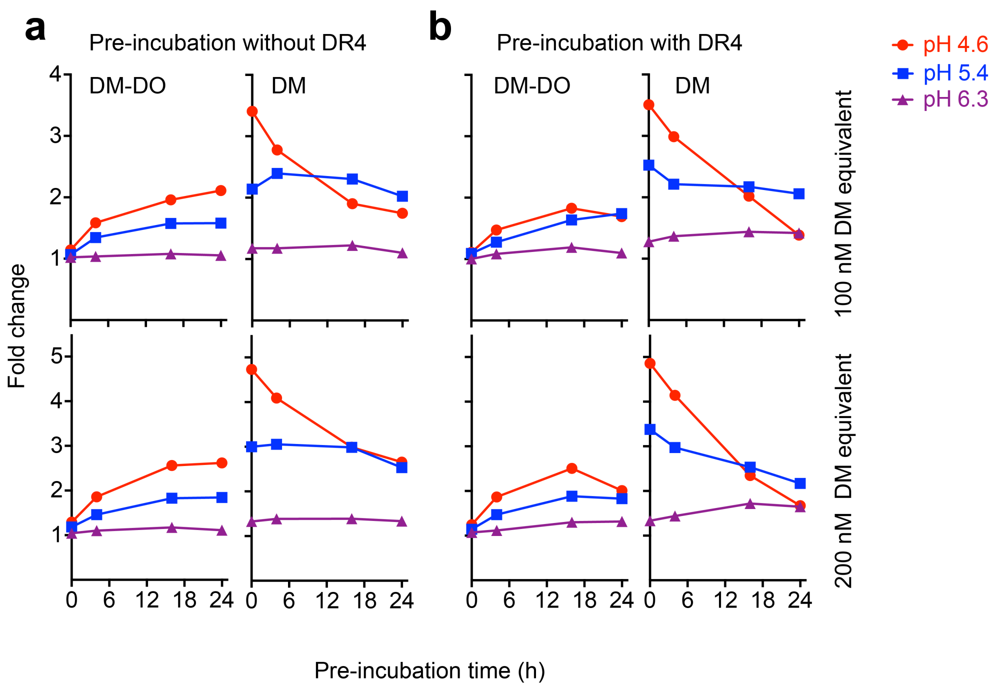

Supplementary Figure 8. Generation of free DM from DM-DO ectodomains is not likely triggered by the competitive binding of soluble DR4 to the DM ectodomain.

Indicated concentrations of DM-DO or DM were pre-incubated (**a**) without or (**b**) with DR4 (5 nM final concentration) in reaction buffer at 37°C for 0, 4, 16, or 24 h, mixed and incubated with (**a**) bio-HA (1.5 μM final concentration) and soluble DR4 (5 nM final concentration) or (**b**) bio-HA only (1.5 μM final concentration) for another 1.5 h before quantitation of DR4-HA. Fold change was calculated as described in Supplementary Figure 7. Data are representative of three experiments.

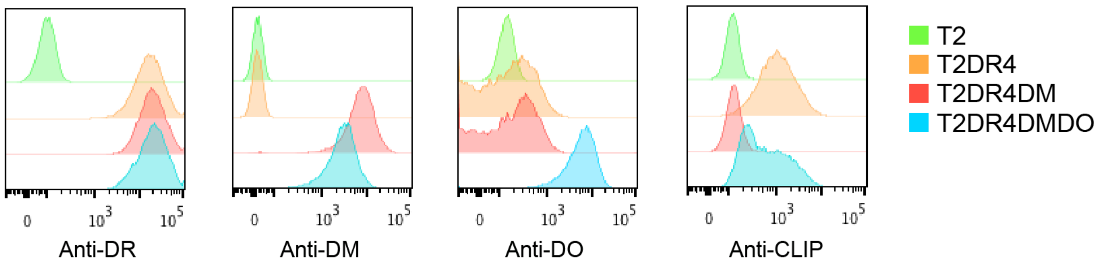

Supplementary Figure 9. Flow cytometric analysis of T2-MHC-II transfectants indicate that T2DR4DMDO and T2DR4 express comparable levels of DR4 and CLIP is a major peptide in complex with these DR4 molecules.

Cells as indicated were fixed and permeabilized before co-staining with DR-, DM-, DO-, and CLIP-specific antibodies, each directly conjugated with a different fluorophore. Overlays of histograms (half offset) are shown. Data are representative of three experiments.

Supplementary Tables

Supplementary Table 1. Intact DO_v after pH 4.6 pulses and untreated DO_v bind DM with similar $K_{D,\ app}$, even though the $K_{D,\ app}$ values for untreated DO_v and DM at pH 4.85 and pH 6.3 are significantly different.

pH	$K_{D,\ app}$ (nM)			
	SPR[a] at 25 °C		SPR at 37 °C	BLI[c] at 25 °C
7.2	9.4		--	11.3
6.3	14.1	**27.5**[b]	12.6	17.0
5.5	21.9		--	26.9
4.85	147.9		239.5	232.6

a. SPR data (**Fig. 2b**) acquired at each concentration of DO_v were fitted to equation ⑦ $[RU] = [RU]_{eq} \times (1- \exp(-k_{obs}\, t))$ (see **Methods**). The resonance units (RU) at equilibrium were plotted against the concentration of DO_v, and fitted to equation ⑧ $[RU]_{eq} = [DO_v][RU]_{max}/(K_{D,\ app} + [DO_v])$ for calculation of $K_{D,app}$.

b. Intact DO_v purified after pH 4.6 pulses was used in the SPR analysis (**Fig. 2f)** and the data were fitted to the above equations for calculation of $K_{D,app}$.

c. BLI data (**Supplementary Fig. 3a**), acquired at a given concentration of DM, were fit to equation $y = y_0 + y_{eq} \times (1- \exp(-k_{obs}\, t))$, as described previously[9], where y_0 was set to 0 unless there was significant non-specific baseline noise. The wavelength shifts (y) at equilibrium were plotted against the concentration of DM and fitted to equation $y_{eq} = [DM]y_{max}/(K_{D,\ app} + [DM])$ for calculation of $K_{D,\ app}$.

--: not determined

Supplementary Table 2. Expression of class II proteins in transfected cell lines.

Cell lines	Expression of class II alleles (including DM and DO)	Antibiotic resistance	Sources
T2	None	None	Ref [1]
T2DM	DM	Puromycin	Ref [10]
T2DR4	DR4	Geneticin*	Refs [2, 10]
T2DR4DM	DR4, DM	Geneticin, puromycin	Refs [2, 10]
T2DR4DMDO	DR4, DM, DO	Geneticin, puromycin, zeocin	This work
T2DQ6	DQ6	Geneticin	This work
S2	None	None	
S2-sDR4	Recombinant DR4 ectodomain (rDR4)	Geneticin	Ref [11]
S2-sDR1	Recombinant DR1 ectodomain	Geneticin	Refs [11, 12]
S2-sDR3	Recombinant DR3 ectodomain	Geneticin	Ref [13]
S2-sDQ6	Recombinant DQ6 ectodomain	Geneticin	This work
S2-sDQ1	Recombinant DQ1 ectodomain	Geneticin	This work
S2-sDM-Avi	DM ectodomain (Avi indicates avitag at the c-terminus)	Geneticin	Refs [8, 9]
S2-szDOv-Avi	Recombinant DOv ectodomain (with αP11A mutation)	Geneticin	Refs [8, 9]
S2-sDMDO-Avi	Recombinant DM and DO ectodomains (mostly in the complex form of DM-DO)	Geneticin	Refs [8, 9]
S2-sDOv(βS63C)	Recombinant DO mutant ectodomain (with αP11A and βS63C mutations)	Geneticin	Ref [9]
S2-sDOv(αR80C)	Recombinant DO mutant ectodomain (with αP11A and αR80C mutations)	Geneticin	Ref [9]

* 0.5-1mg/ml geneticin (Life Technologies), 0.5-1 μg/ml puromycin (Sigma), and 50-100 μg/ml zeocin, respectively, were added into corresponding cell cultures to maintain the selection of stable transfactants.

Supplementary Table 3. Oligonucleotides used in PCR reactions for the construction of plasmids.

Plasmids		Primers	RE recognition sites
pBudCD4.1-DOA/DOB			
DOA	SN*	5'-TGATGTCGACAAAGCACCAGAGTGTAATGG-3'	SalI
	ASN**	5'-ATCTGGATCCTTACCTGGGGACACTGGACA-3'	BamHI
DOB	SN	5'-TTATGCGGCCGCATGGGTTCTGGGTGGGTCCCCT-3'	NotI
	ASN	5'-ATCTAGATCTTTAGCATGACTGAGGGAGCA-3'	BglII
pBMN-ZIN-neo-DQ6A			
	SN	5'-CGGGATCCATGATCCTAAACAAAGCTCTGCTGCTGGG-3'	BamHI
	ASN	5'-ACGCGTCGACTCACAATGGC- CCTTGGTGTCTGGA-3'	SalI
pBMN-ZIN-neo-DQ6B			
	SN	5'-CCGGAATTCATGTCTTGGAAGAAGGCTT-3'	EcoRI
	ASN	5'-ACGCGTCGACTCAGTGCAGAAGCCCTTT-3'	SalI
pRmHa3-sDQ6A-Fos			
	SN	5'-GTCAGAATTCATGATCCTAAACAAAGCTCTGCT-3'	EcoRI
	ASN	5'-TACTGTCGACAGTCTCTGTGAGCTCTGACA-3'	SalI, SacI
Fos	SN	5'-ATGTGAGCTCACAGAGACTGGAGGTGGCGGTTCACTGGAAGTTCTG TTCCAGGGGCCCGGAGGCGGTTTAACTGATACACTC-3'	SacI
	ASN	5'-ATGTGTCGACTTAGTGCCACTCGATCTTCTG-3'	SalI
pRmHa3-sDQ6B-Jun			
	SN	5'-TATGTCCGGA*CCTGTGAGCAAGATGAGAATGGCCACCCCCCTGCTC ATGCAGGCT*GGAGGTGGTGGCTCACTAGTGCCA-3'	BspEI
Jun	ASN	5'-TACTGTCGACGGCTCTGAAGGTACCCTTGCTCTGGGCAGATTCAG-3'	SalI, KpnI
	SN	5'-ATGTGGTACCGGAGGTGGCGGTTCACTGGAAGTTCTGTTCCAAGG TCCTGGAGGTGGTCGCATCGCCCGGCTCGA-3'	KpnI
	ASN	5'-GCTGGTCGACTCAGCAATGGTTCATGACTTTCTGT-3'	SalI
pRmHa3-sDQ1A			
	SN	5'-CCGGAATTCATGATCCTAAACAAAGCTCTGCTGCTGGGGGCC-3'	EcoRI
	ASN	5'-ACGCGTCGACCTCTGTGAGCTCTGACATAGGGGCTGGAATCT-3'	SalI
pRmHa3-sDQ1B			
	SN	5'-CCGGAATTCATGTCTTGGAAGAAGTCTTTGCGGATCCCCGG-3'	EcoRI
	ASN	5'-ACGCGTCGACCTTGCTCTGGGCAGATTCAGACTGAGC-3'	SalI

* SN: sense primer;

** ASN, antisense primer;

Underlined sequences are RE recognition sites. *Italic indicates* a sequence encoding $CLIP_{87-101}$. Sequences in gray have homology to the corresponding class II cDNAs.

Supplementary Table 4. pH of different reaction buffers measured at the indicated temperature.

Buffers	4 °C	25 °C	37 °C
Acetic acid/sodium acetate	4.69	4.58	4.57
Citric acid/sodium citrate	4.66	4.56	4.52
	4.81	4.72	4.70
	4.88	4.82	4.82
	–	4.88	4.88
	5.08	4.99	4.99
	–	5.07	5.10
	5.18	5.15	5.16
	–	5.23	5.25
	–	5.30	5.31
	5.50	5.41	5.43
Monosodium phosphate/disodium phosphate	6.37	6.33	6.36
	7.20	7.13	7.16

–: not determined.

References

1. Salter, R.D., Howell, D.N. & Cresswell, P. Genes regulating HLA class I antigen expression in T-B lymphoblast hybrids. *Immunogenetics* **21**, 235-246 (1985).
2. Rinderknecht, C.H. et al. DM influences the abundance of major histocompatibility complex class II alleles with low affinity for class II-associated invariant chain peptides via multiple mechanisms. *Immunology* **131**, 18-32 (2010).
3. Hou, T. et al. An insertion mutant in DQA1*0501 restores susceptibility to HLA-DM: implications for disease associations. *J Immunol* **187**, 2442-2452 (2011).
4. Glazier, K.S. et al. Germinal center B cells regulate their capability to present antigen by modulation of HLA-DO. *J Exp Med* **195**, 1063-1069 (2002).
5. Hammond, C. et al. The tetraspan protein CD82 is a resident of MHC class II compartments where it associates with HLA-DR, -DM, and -DO molecules. *J Immunol* **161**, 3282-3291 (1998).
6. Doebele, R.C., Busch, R., Scott, H.M., Pashine, A. & Mellins, E.D. Determination of the HLA-DM interaction site on HLA-DR molecules. *Immunity* **13**, 517-527 (2000).
7. Spits, H. et al. HLA-DC antigens can serve as recognition elements for human cytotoxic T lymphocytes. *Eur J Immunol* **14**, 299-304 (1984).
8. Guce, A.I. et al. HLA-DO acts as a substrate mimic to inhibit HLA-DM by a competitive mechanism. *Nat Struct Mol Biol* **20**, 90-98 (2013).
9. Yoon, T. et al. Mapping the HLA-DO/HLA-DM complex by FRET and mutagenesis. *Proc Natl Acad Sci U S A* **109**, 11276-11281 (2012).
10. Denzin, L.K., Robbins, N.F., Carboy-Newcomb, C. & Cresswell, P. Assembly and intracellular transport of HLA-DM and correction of the class II antigen-processing defect in T2 cells. *Immunity* **1**, 595-606 (1994).
11. Sloan, V.S. et al. Mediation by HLA-DM of dissociation of peptides from HLA-DR. *Nature* **375**, 802-806 (1995).
12. Busch, R., Reich, Z., Zaller, D.M., Sloan, V. & Mellins, E.D. Secondary structure composition and pH-dependent conformational changes of soluble recombinant HLA-DM. *J Biol Chem* **273**, 27557-27564 (1998).
13. Fallang, L.E. et al. Complexes of two cohorts of CLIP peptides and HLA-DQ2 of the autoimmune DR3-DQ2 haplotype are poor substrates for HLA-DM. *J Immunol* **181**, 5451-5461 (2008).
